# Statistical inference using GLEaM model with spatial heterogeneity and correlation between regions

**DOI:** 10.1038/s41598-022-18775-8

**Published:** 2022-10-05

**Authors:** Yixuan Tan, Yuan Zhang, Xiuyuan Cheng, Xiao-Hua Zhou

**Affiliations:** 1 Department of Mathematics, Duke University, Durham, USA; 2 School of Statistics, Renmin University of China, Beijing, China; 3 Center for Statistical Sciences, Peking University, Beijing, China; 4 Beijing International Center for Mathematical Research, Peking University, Beijing, China; 5 Department of Biostatistics, School of Public Health, Peking University, Beijing, China

**Keywords:** Epidemiology, Statistics

## Abstract

A better understanding of various patterns in the coronavirus disease 2019 (COVID-19) spread in different parts of the world is crucial to its prevention and control. Motivated by the previously developed Global Epidemic and Mobility (GLEaM) model, this paper proposes a new stochastic dynamic model to depict the evolution of COVID-19. The model allows spatial and temporal heterogeneity of transmission parameters and involves transportation between regions. Based on the proposed model, this paper also designs a two-step procedure for parameter inference, which utilizes the correlation between regions through a prior distribution that imposes graph Laplacian regularization on transmission parameters. Experiments on simulated data and real-world data in China and Europe indicate that the proposed model achieves higher accuracy in predicting the newly confirmed cases than baseline models.

## Introduction

The outbreak of coronavirus disease 2019 (COVID-19) has impacted all aspects of the world significantly for a long. As of 26 Oct 2021, over 243 million confirmed cases of COVID-19 have been reported, including over 4 million deaths^1^. Therefore, it is essential to study the spread of COVID-19 for better prediction and prevention of the disease. This paper proposes a new stochastic dynamical model that can describe different spread patterns of COVID-19 in multiple regions. We also develop an algorithm to estimate the corresponding transmission parameters and their posterior distributions. Our model is inspired by the Global Epidemic and Mobility (GLEaM) model proposed in Ref.^2^. GLEaM is a stochastic dynamic model that depicts the spread of epidemics, integrating multiple data layers. The GLEaM model involves 3362 subpopulations in 220 countries obtained from Voronoi tessellation, centered around major airports. These subpopulations are connected by a multi-layered mobility network composed of processes from short-range commuting between nearby subpopulations to international flights. In each subpopulation, the transmission of epidemics is modeled by a variant of an Susceptible-Exposed-Infected-Removed (SEIR) compartmental model^3^. Please see “Review of the SEIR model and GLEaM model” for a more detailed review of the SEIR model and GLEaM model.

In the vast majority of GLEaM’s applications^4–11^, the parameters are estimated based on Ref.^12^. Reference^12^ performed the maximal likelihood analysis of the reproduction number $R_0$ in the seed region, Mexico. For each value of the reproduction number $R_0$, the method generated the distribution of the arrival time of the influenza A(H1N1) in 12 countries produced by $2\times 10^3$ GLEaM simulations. Then, the optimal reproduction number $R_0$ was chosen by maximizing the likelihood function of arrival time. Reference^12^ and subsequent works following its settings^4–11^ assumed that the epidemic was seeded from one region and the transmission parameters or the other parameters (like the introduction date and location) were estimated through the maximal likelihood analysis of arrival time or other events. In particular, the method in Ref.^12^ was adopted in Ref.^6^ to estimate the posterior distribution of the reproduction number $R_0$ of COVID-19, which was assumed to be uniform for all subpopulations at all times. However, this setting is unsuitable for the current scenario of the COVID-19 pandemic since COVID-19 has lasted for a long time, and the community transmission has been widespread in most countries in the world^13^. To model the spread of COVID-19, both spatial and temporal heterogeneity of the transmission parameters are needed, rather than directly modeling the reproduction number $R_0$ solely as a periodic function of time as in Ref.^12^. This is because the social behaviors, containment measures, medical conditions, and other elements that affect the spread of COVID-19 may vary among different countries and over time.

Recently, Reference^14^ improved the inference method in Ref.^12^ based on the GLEaM model, by involving spatial heterogeneity. Specifically, Ref.^14^ estimated the initially infected individuals in each subpopulation through microblogging data from Twitter and also estimating the reproduction number $R_0$ for USA, Italy, and Spain separately. However, international travel was not considered in this study, and GLEaM was applied to each of the aforementioned countries (as an isolated systems) independently. The transmission rates in all subpopulations of these countries were again presumed to be homogeneous. Furthermore, Ref.^14^ also assumed that the initially infected individuals for each subpopulation were proportional to the total number of Twitter users in that subpopulation. Thus it still assumed that the severity of the pandemic at the initial outbreak of COVID-19 was uniform over the country, which is not the case for COVID-19.

In addition to the abovementioned issues, other potential concerns exist in applying GLEaM to model the spread of COVID-19. As mentioned in the last section of Ref.^15^, GLEaM can be used to simulate the spread of the epidemic under normal conditions since it uses the “steady-state” mobility data around the world. However, since the outbreak of COVID-19, the social order has been disrupted, and travel has been restricted in most countries. Thus GLEaM might not work well with its multi-layered mobility networks. Furthermore, the estimate of parameters using GLEaM is based on a large number of simulations to explore the space of parameters, which may potentially take much computational time^2^ when the epidemic parameters to be estimated are spatially heterogeneous. In addition, although the social behavior, medical conditions, and other factors that affect the spread of COVID-19 may vary among different regions, these factors for regions that are geographically close or have similarities in other aspects still bear some resemblance. Hence, the transmission rates for COVID-19 should not only have their own heterogeneity but also be correlated to each other. To the best of our knowledge, neither of the features is reflected in GLEaM or most of its applications.

As the consequences of the possible constraints of GLEaM described above, most of the papers using GLEaM to model the epidemics mainly focus on estimating only the transmission parameter in the seed region at the very beginning of the outbreak. However, for the current long-lasting spread of the COVID-19 pandemic all over the globe, the spatial and temporal heterogeneity of the transmission parameters is needed to be taken into full consideration.

**Figure 1:**
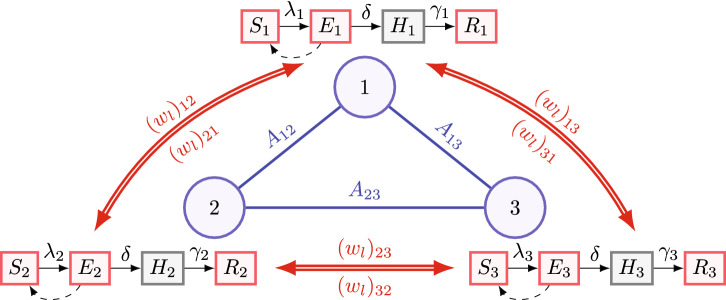
A diagram illustrating the model proposed in this paper. The example includes three regions, marked by circles in blue and indexed by 1, 2, and 3. For $k,j\in \{1,2,3\}$ and $k\ne j$, $A_{kj}$ on the edge (*k*, *j*) represents the similarity between regions *k* and *j*. The square nodes associated with each region denote the compartments for each region, including susceptible, exposed, hospitalized, and removed compartments. The arrows connecting square nodes denote the transition between compartments in each region, and the details can be found in “Model description”. Note that $\lambda _1,\lambda _2,$ and $\lambda _3$, the transmission parameters in the three regions, are allowed to be spatially heterogeneous. The double arrows in red denote the transportation between three regions on the *l*-th day. For $k,j\in \{1,2,3\}$ ($k\ne j$), $(w_l)_{kj}$ represents the total transportation volume from region *k* to region *j* on the *l*-th day.

In this paper, we propose a new stochastic model that incorporates transportation between regions and at the same time enables spatial and temporal heterogeneity of transmission parameters. We model *n* regions as a graph having *n* nodes, and the transportation pattern between the regions is encoded as *n*-by-*n* matrices. Our graphical model of epidemic dynamics is a general abstract one motivated by and simplified from the GLEaM framework. Figure 1 shows a diagram of the proposed model. In contrast to most applications of GLEaM, which mainly focus on the initial outbreak, our proposed model is able to model the long-lasting spread of epidemics. For the inference of model parameters, we introduce an optimization algorithm that utilizes the correlation between districts. Furthermore, the posterior distribution of parameters is estimated by an Markov Chain Monte Carlo (MCMC) sampling procedure, where we set the initial value of the Markov Chain as the optimal parameter obtained by the optimization algorithm. This approach can potentially accelerate the convergence of MCMC sampling.

In summary, the main contributions of our paper are:

- We propose a new stochastic model to describe the epidemic’s long-lasting spread, allowing spatial and temporal heterogeneity of transmission parameters and transportation between districts.
- Based on the proposed model, we also design an algorithm that first makes inference for the parameters through a two-step procedure and then estimates the posterior distribution efficiently by MCMC sampling with the estimated parameters as the initial points. The parameter inference combines the information of correlation between districts, which is equivalent to imposing graph Laplacian regularization on the transmission parameters.
- We compare the performance of the proposed model with the baseline models on both simulated and real-world data.
  - For the simulated data, the results show that combining heterogeneity and transportation into the model helps improve the performance of trajectory prediction and parameter estimation. Moreover, our inference algorithm that integrates the correlation of districts leads to further improvement in predicting the future trajectories.
  - For the real-world data in China and Europe, the proposed model outperforms the baselines in trajectory prediction.

A strength of the proposed model resides in introducing spatial and temporal heterogeneity of transmission parameters. We compare with more related works and comment on the differences and relations in “More related works”. Datasets used in this paper are publicly available at Refs.^16–18^. Our work focuses on the methodology development and we aim at a new stochastic dynamic model that is generally applicable.

We list the default notations and parameters used throughout the paper in Table 1. The rest of the paper is structured in the following way: In “Methods”, we introduce the stochastic dynamic model and the corresponding inference algorithm. In “Experimental results for simulated data”, we compare the performance of trajectory prediction and parameter estimation of the models with or without mobility, heterogeneity, and using correlation information in the inference part for the simulated data. Section “Experimental results on COVID-19 data” describes the real-world data used in this paper, and presents the results and findings of applying the proposed model to the COVID-19 data in China and Europe. We discuss the limitations and possible extensions in “Discussion”.

### Review of the SEIR model and GLEaM model

In this section, we provide a more detailed introduction to the SEIR model and the GLEaM model so as to provide a background of our study and augment the following context.

To depict the evolution of the epidemics, Ref.^19^ proposed the celebrated Susceptible-Infected-Removed (SIR) model and characterized the development of the pandemic with a deterministic ordinary differential equation (ODE). There are many extensions of the SIR model, including the Susceptible-Exposed-Infected-Removed (SEIR) model for diseases with a latent period, the Susceptible-Infected-Susceptible(SIS) model for diseases that do not gain immunity after recovery, etc.

These deterministic transmission models are constructed under certain assumptions, including that the population is large, closed, and homogeneous. Due to the random nature of the transmission process, many stochastic dynamic models are developed^20–22^. Under certain rather generalized conditions, the deterministic models can be seen as the mean-field equations of the corresponding stochastic processes. However, this approximation may not hold when the size of the outbreak has not grown up to the same order of the total population, which is the case in many applications^23^. More details can be found in Ref.^24^ and the references therein.

The Global Epidemic and Mobility (GLEaM) model proposed in Ref.^2^ used a meta-population scheme which balanced between the agent-based stochastic models and the deterministic compartmental models. Specifically, Ref.^2^ adapted a high-resolution population database that divided the surface of the earth with cells of 15 min $\times$ 15 min of arc, and then used Voronoi tessellation to assign each cell to one of the major airports around the world. The obtained subdivisions were then called subpopulations.

The stochastic dynamic in the subpopulations was then coupled with two layers of mobility flows apart from the infection dynamic within each subpopulation. The first layer was the worldwide airport network between the airports in the subpopulations, which could be seen as a weighted graph whose edges represented the number of passengers between each pair of airports. This layer was integrated into the model through stochastic transportation between subpopulations. The second layer was the commuting network that connected subpopulations graphically close. This layer was integrated through being used to compute the effective population and infection in each subpopulation. More details can be found in Ref.^2^.

### More related works

Several recent works also involved different levels of heterogeneity in their models in various ways. Reference^25,26^ utilized randomness in reproduction numbers to reflect the heterogeneity of the population, using plate model with Bayesian method and heterogeneous well-mixed theory^27^ with age-of-infection method^19^, respectively. References^28,29^ used functional data analysis tools. Specifically, Ref.^28^ captured two different epidemic patterns in different regions of Italy using the probKMA algorithm Refs.^30^, and^29^ revealed different patterns of the epidemic across countries with functional principle component analysis. In addition, Refs.^31–35^ adapted SEIR / Susceptible-Exposed-Infected (SEI) / Susceptible-Infected (SI) compartmental models similar to this paper. Among these works,^31–33^ considered heterogeneity in the aspects of age groups, social links, and vaccination status separately. References^34,35^ bore more similarity with our paper since they also allowed transmission parameters to be spatially heterogeneous and involved transportation between different regions. However, Ref.^34^ only considered intracounty data, and the transportation was used to compute the effective size of compartments and did not affect the dynamic model. Furthermore, the transmission rates in Ref.^34^ were determined by an SDE whose parameters were to be fitted. Therefore, Ref.^34^ focused on a different scope from our study. The settings of compartments in Ref.^35^ were more realistic than the one considered in our paper by considering reporting rates. Nevertheless, compared with the model and inference algorithm described in “Methods”, transmission rates in Ref.^35^ did not have temporal heterogeneity or correlation with each other. Both Refs.^34,35^ used the Ensemble Kalman Filter, which samples particles in the state space according to the prior distribution and obtains the posterior distribution in the process of moving particles at each time step. This might be computationally less efficient than directly applying MCMC according to the posterior distribution with the initial point maximizing the posterior distribution, as implemented in this paper.

## Methods

### Ethics statement

The medical record data in China and Europe used in this paper are publicly available and can be found on the official websites of the National Health Commission of the People’s Republic of China^16^, the Chinese Center for Disease Control and Prevention^17^, and European Centre for Disease Prevention and Control^18^. The collection of data is performed in compliance with local government regulations. More details about data sources can be found in “Data sources”.

### Model description

#### Compartmental model over multiple regions

In GLEaM^2^ and other epidemic models involving transportation^34,35^, the whole area is usually divided into subdivisions. For example, the GLEaM model divides the total area of 220 countries into over 3300 subpopulations centered around major airports and^34^ divided Milwaukee County and Dane County in the state of Wisconsin into several regions. In this paper, we consider abstract subdivisions in the whole area, which will be referred to as “regions” hereinafter until further specifications in the later experiment sections. We denote *n* as the number of regions.

In our model, we use continuous time $t \in [0,T]$, where it is assumed that the evolution of the epidemic lasts within a period of *T* time units. The unit of time is fixed as one day throughout this paper. Note that when we introduce the transportation model in below the traveling matrix is assumed to be constant within each day, and the observed data is also collected on a daily basis. Thus we will use notation of discrete time (days) from $1,\dots ,T$ hereinafter, however, the evolution dynamic itself is modeled over continuous time.

For each region, we consider the following epidemiology compartments adapted from the SEIR model:

- $$S_k(t)$$: Susceptible.
- $E_k(t)$: Exposed and infectious.
- $$H_k(t)$$: Hospitalized.
- $R_k(t)$: Removed (recovered or dead).

The subscript *k* of the states denotes that they belong to the *k*-th region, and the dependence on the continuous time *t* is addressed through expressing the states as functions of $t\in [0, T]$.

At time *t*, we use $N_k(t) = S_k(t) + E_k(t) + H_k(t) + R_k(t)$ to denote the total population in the *k*-th region. The population $N_k(t)$ is allowed to be time-varying due to the inter-region mobility, especially for the days before the implementation of travel restrictions. However, since the traveling volume is not comparable to the total population in a region, the fluctuation of the total population in a region is not obvious. In this paper, we assume that $N = \sum _{k=1}^n N_k(t)$ keeps constant over time, which means that we consider a closed system, where exported/imported cases are not considered. However, it is worth noting that we do allow the transportation of active virus carriers between regions within our system. We remark in advance that this assumption is reasonable for the real-world data sets considered in this paper. From January to February 2020, strict international travel restrictions were imposed in China. While for data in Europe, from May to August 2020, the local spread of the epidemic has reached a relatively high level, and the imported cases were not comparable to the indigenous cases. We also denote $(N_a)_k(t) = S_k(t) + E_k(t) + R_k(t)$ as the total population that are permitted to move in the *k*-th region, excluding the hospitalized ones.

#### Transportation between regions and the stochastic model

Transportation plays an essential role in the spread of COVID-19. Actually, Refs.^36,37^ indicated that the travel restrictions were remarkably important in mitigating the transmission of COVID-19, especially in the early stage of the pandemic. Recently, as detailed in Ref.^38^, the Omicron variant had spread to 110 countries and had become dominant in many of them by 22 December 2021, only one month after its first report from South Africa on 24 November 2021. This motivates us also to take transportation into consideration in this paper. In our model, we introduce the transportation between regions via a traveling matrix, which is similar to the notation in the GLEaM model^2^. Specifically, we denote $(w_l)_{kj}$ as the traveling volume from region *k* to *j* on the *l*-th day ($l=1,\dots ,T$). Then the traveling matrix $W_l$ on the *l*-th day can be written as

2.1 $$\begin{aligned} W_l=\begin{pmatrix} (w_l)_{11} &{} (w_l)_{12} &{} \cdots &{} (w_l)_{1n}\\ (w_l)_{21} &{} (w_l)_{22} &{} \cdots &{} (w_l)_{2n}\\ \vdots &{} \vdots &{} \ddots &{} \vdots \\ (w_l)_{n1} &{} (w_l)_{n2} &{} \cdots &{} (w_l)_{nn}\\ \end{pmatrix}. \end{aligned}$$

Given the transportation matrix, we describe in below the stochastic model of the dynamic of the compartments over *n* regions, denoted as $\{ (S_k(t), E_k(t), H_k(t), R_k(t)), k =1 \dots , n \}$, where $t\in [0,T]$ is the continuous time, and the variables $S_k(t)$, $E_k(t)$, $H_k(t)$, and $R_k(t)$ take integer values from 0 to *N*. The proposed stochastic model is illustrated in Fig. 1.

- Transmission in the *k*-th region: A case from $E_k(t)$ chooses an individual from $N_k(t)$ randomly at Poisson rate $\lambda _k$ ($\{\lambda _k\}_{k=1}^n$ are allowed to be spatially heterogeneous), and the individual chosen is infected if it is of state $S_k(t)$. Note that this is different from traditional SEIR models, since we assume that for the COVID-19 case, the pre-symptomatic patients from $E_k(t)$ can be contagious.
- Hospitalization in the *k*-th region: Each individual in $E_k(t)$ will be hospitalized with Poisson rate $\delta$.
- Recovery or death in the *k*-th region: Each individual in $H_k(t)$ will transfer into $R_k(t)$ with Poisson rate $\gamma _k$. The rate $\gamma _k$ owns spatial heterogeneity due to the uneven distribution of medical resources.
- Transportation between regions: At the end of the *l*-th day, all the individuals in region *k* except the ones in $H_k(l)$ have the same probability of traveling from region *k* to region *j*, and the total traveling volume from region *k* to region *j* is $(w_l)_{kj}$. We assume that there are no transmissions happening during the transportation between regions. If we denote $\{\xi _{kj}^{[S,l]}, \xi _{kj}^{[E,l]}, \xi _{kj}^{[R,l]}\}$ as the number of people transported from $\{S_k(l), E_k(l), R_k(l)\}$ to $\{S_j(l), E_j(l), R_j(l)\}$ at the end of the *l*-th day, then $\{\xi _{kj}^{[S,l]}, \xi _{kj}^{[E,l]}, \xi _{kj}^{[R,l]}\}$ follows a multinomial distribution. Specifically,
  2.2 $$\begin{aligned} P\left( \left\{ \xi _{kj}^{[S,l]}, \xi _{kj}^{[E,l]}, \xi _{kj}^{[R,l]}\right\} \right) = \frac{\left( (w_l)_{kj}\right) !}{\left( \xi _{kj}^{[S,l]}\right) !\left( \xi _{kj}^{[E,l]}\right) !\left( \xi _{kj}^{[R,l]}\right) !} \left( \frac{S_k(l)}{(N_a)_k(l)}\right) ^{\xi _{kj}^{[S,l]}} \left( \frac{E_k(l)}{(N_a)_k(l)}\right) ^{\xi _{kj}^{[E,l]}} \left( \frac{R_k(l)}{(N_a)_k(l)}\right) ^{\xi _{kj}^{[R,l]}}, \end{aligned}$$
  with $$\xi _{kj}^{[S,l]}+\xi _{kj}^{[E,l]}+\xi _{kj}^{[R,l]}=(w_l)_{kj}.$$

As a consequence, $N_k(t)$ is a piece-wise constant function of *t* which only changes at the end of each day. Specifically, for any time $t \ge 0$,

2.3 $$\begin{aligned} N_k(t) = N_k(0) + \sum _{l=1}^{\lfloor t\rfloor } \sum _{i=1}^n ((w_l)_{ik} - (w_l)_{ki}). \end{aligned}$$

A more comprehensive stochastic dynamic model has been previously developed in Ref.^24^. However, the work did not consider transportation between regions, which is a focus of this study.

**Table 1:** List of notations and parameters.

Notation	Description	Notes
*n*	Total number of regions	
*T*	Total number of days	
$S_k(t)$	Susceptible individuals in the *k*-th region at time *t* $1\le k\le n,t\in [0,T]$,	The same range of *k* and *t* also holds below
$E_k(t)$	Exposed individuals in the *k*-th region at time *t*	$\{E_k(0)\}_{k=1}^n$ need to be inferred
$H_k(t)$	Hospitalized individuals in the *k*-th region at time *t*	$\{H_k(0)\}_{k=1}^n$ need to be inferred
$R_k(t)$	Removed individuals in the *k*-th region at time *t*	$R_k(0)=0,\forall k$
$N_k(t)$	Total individuals in the *k*-th region at time *t*	$\sum _{k=1}^n N_k(t)$ is assumed to be constant over time
$W_l$	Traveling volume matrix on the *l*-th day	$(W_l)_{ij} = (w_l)_{i,j}, l\in \{1,\dots ,T\}$
$\widetilde{S}_k(t)$	Deterministic counterpart of $S_k(t)$,	Other compartments follow the same
	Determined by (2.4)	Convention of notations, $t\in [0,T]$
$(\widetilde{C_a})_k(t)$	Accumulated confirmed cases determined by (2.4)	$t\in [0,T]$
$(\widetilde{R_a})_k(t)$	Accumulated removed cases determined by (2.4)	$t\in [0,T]$
$(C_a)_k(i)$	Accumulated confirmed cases on the *i*-th day	$i\in \{1,\dots ,T\}$
$(R_a)_k(i)$	Accumulated removed cases on the *i*-th day	$i\in \{1,\dots ,T\}$
$(\widetilde{\Delta C_a})_k(i)$	Newly confirmed cases determined by (2.4) on the *i*-th day	$i\in \{1,\dots ,T\}$
$(\Delta C_a)_k(i)$	Newly confirmed cases on the *i*-th day	$i\in \{1,\dots ,T\}$
$\lambda _k$	Infection rate in the *k*-th region	$\{\lambda _k\}_{k=1}^n$ need to be inferred and are allowed to be time-varying
$\delta$	Inverse of average incubation period	$\delta$ is prefixed as 0.14
$\gamma _k$	Inverse of average removed time in the *k*-th region	$\{\gamma _k\}_{k=1}^n$ need to be inferred
$\Theta$	Set of parameters to be estimated	
*W*	The matrix characterizing the proximity between regions	
*d*	Total number of groups that *n* regions are divided into	
$D_m$	The *m*-th group of regions	$1\le m\le d$
$\mu$	Penalty factor of Graph Laplacian regularization	
$\beta$	Parameter reducing regularization between inter-group regions	$\beta \in (0,1)$
*A*	Affinity matrix constructed by *W*, $\mu$, and $\beta$ as in (2.12)	
$\sigma$	Parameter in the prior (2.10)	

#### Differential equation with spatial heterogeneity

Following Refs.^39,40^, we derive the corresponding mean-field differential Eq. (2.4) of the stochastic dynamic introduced in “Transportation between regions and the stochastic model”, which is continuous in time, and the compartments $(\widetilde{S}_k(t), \widetilde{E}_k(t), \widetilde{H}_k(t), \widetilde{R}_k(t))$ take real values.

2.4 $$\begin{aligned} {\left\{ \begin{array}{ll} \dfrac{d\widetilde{S}_k(t)}{dt} = -\lambda _k\dfrac{\widetilde{S}_k(t)\widetilde{E}_k(t)}{\widetilde{N}_k(t)} + \sum _{i\ne k}\bigg [(w_{\lceil t\rceil })_{ik}\dfrac{\widetilde{S}_i(t)}{(\widetilde{N_a})_i(t)} - (w_{\lceil t\rceil })_{ki}\dfrac{\widetilde{S}_k(t)}{(\widetilde{N_a})_k(t)}\bigg ] \\ \dfrac{d\widetilde{E}_k(t)}{dt} = \lambda _k\dfrac{\widetilde{S}_k(t)\widetilde{E}_k(t)}{\widetilde{N}_k(t)} -\delta \widetilde{E}_k(t)+ \sum _{i\ne k}\bigg [(w_{\lceil t\rceil })_{ik}\dfrac{\widetilde{E}_i(t)}{(\widetilde{N_a})_i(t)} - (w_{\lceil t\rceil })_{ki}\dfrac{\widetilde{E}_k(t)}{(\widetilde{N_a})_k(t)}\bigg ]\\ \dfrac{d\widetilde{H}_k(t)}{dt} = \delta \widetilde{E}_k(t)-\gamma _k \widetilde{H}_k(t)\\ \dfrac{d\widetilde{R}_k(t)}{dt} = \gamma _k \widetilde{H}_k(t)+ \sum _{i\ne k}\bigg [(w_{\lceil t\rceil })_{ik}\dfrac{\widetilde{R}_i(t)}{(\widetilde{N_a})_i(t)} - (w_{\lceil t\rceil })_{ki}\dfrac{\widetilde{R}_k(t)}{(\widetilde{N_a})_k(t)}\bigg ] \\ {\dfrac{d\widetilde{N}_k(t)}{dt}=\sum _i ((w_{\lceil t\rceil })_{ik} - (w_{\lceil t\rceil })_{ki})} \\ \dfrac{d(\widetilde{C_a})_k(t)}{dt} = \delta \widetilde{E}_k(t) \\ \dfrac{d(\widetilde{R_a})_k(t)}{dt} = \gamma _k \widetilde{H}_k(t) \end{array}\right. } \end{aligned}$$

The first four equations of (2.4) describe the evolution of the $S_k(t), E_k(t), H_k(t), R_k(t)$ in the deterministic version of our model, which is governed by the transition dynamic explained in “Transportation between regions and the stochastic model”. The fifth equation characterizes the deterministic total population, which is a piece-wise linear function of time *t* (since the traveling volume is a piece-wise constant function of *t*) and coincides with $N_k(t)$ expressed as in (2.3) when *t* takes integer values. The last two equations depict the evolution of accumulated confirmed and removed cases, denoted by $(\widetilde{C_a})_k(t)$ and $(\widetilde{R_a})_k(t)$ respectively, in the deterministic model. It is worth noting that in the calculation of $(\widetilde{C_a})_k(t)$ and $(\widetilde{R_a})_k(t)$, each case is only accounted for once. In (2.4), $\widetilde{S}_k(t)$ is the deterministic counterpart of $S_k(t)$ and the same for $\widetilde{E}_k(t), \widetilde{H}_k(t), \widetilde{R}_k(t), (\widetilde{N_a})_k(t)$.

Furthermore, we assume that the accumulated confirmed and removed cases are available from data on a daily basis, which are denoted as $\{(C_a)_k(i)\}_{i=1}^T$ and $\{(R_a)_k(i)\}_{i=1}^T$, respectively. We also assume that $R_k(0)$ is 0, while $E_k(0)$ and $H_k(0)$ are left to be inferred for each $k=1,\dots ,n$. For inference of parameters, we further denote $(\widetilde{\Delta C_a})_k(i) = (\widetilde{C_a})_k(i) - (\widetilde{C_a})_k(i-1)$ as the deterministic newly confirmed cases on the *i*-th day determined by (2.4), and $(\Delta C_a)_k(i)$ as the newly confirmed cases computed from data, namely $(\Delta C_a)_k(i) = (C_a)_k(i) - (C_a)_k(i-1)$, $k=1,\dots ,n, i=2,\dots ,T$. The same convention holds for the definitions of $(\widetilde{\Delta R_a})_k(i)$ and $(\Delta R_a)_k(i)$. Note that the data $\{(C_a)_k(i), (R_a)_k(i), (\Delta C_a)_k(i), (\Delta R_a)_k(i)\}_{i=1}^T$ are random in nature.

Note that the model and the inference algorithm described below can be applied to estimate parameters as long as $\{(C_a)_k(i),k=1,\dots ,n\}_{i=1}^T$, $\{(R_a)_k(i),k=1,\dots ,n\}_{i=1}^T$, and $\{W_l\}_{l=1}^T$ are available. The availability of $\{(C_a)_k(i),k=1,\dots ,n\}_{i=1}^T$ and $\{(R_a)_k(i),k=1,\dots ,n\}_{i=1}^T$ is required in many works that use the SEIR model to estimate transmission rates of the epidemic^41–43^, and the transportation network is also used in GLEaM^2^ and its applications. However, in contrast to the works based on GLEaM^4–6,12,15^, we allow parameters to possess both spatial and temporal heterogeneity, and further utilize the correlation between regions in the inference of parameters. We remark that the spatial and temporal heterogeneity is reflected in the fact that the transmission parameters $\{\lambda _k\}$ are allowed to vary in both space and time in our model. The temporal heterogeneity is introduced in more detail for the real-world data in “Model extension by allowing time-varying parameters”.

### Estimation of model parameters

Based on the model described in “Model description”, the parameters that need to be specified are $\delta$ and $\Theta : = \{E_k(0), H_k(0), \lambda _k, \gamma _k\}_{k=1}^n$. Using a simplification in Remark 1, we prefix the parameter $\delta$, and estimate the rest in a two-step procedure to be described in this section. As a brief summary,

- Step 1. We first make inference for $\{\gamma _k\}_{k=1}^n$ by maximizing the likelihood of the observed newly removed cases. Details in “[Sec Sec11]”.
- Step 2. After the estimation of $\{\gamma _k\}_{k=1}^n$, ${\overline{\Theta }} := \{E_k(0), H_k(0), \lambda _k\}_{k=1}^n$ are then estimated by maximizing the posterior probability, where we introduce a prior distribution combining the information of correlation between regions. Details in “[Sec Sec12]”.

Finally, in the end of Step 2, we introduce an MCMC sampling approach to estimate the marginal posterior distributions of ${\overline{\Theta }}$. This provides information about the uncertainty of the estimated parameters, like $\lambda _k$, which are of scientific interest. We summarize the two-step procedure in this section together in Algorithm 1.

#### Remark 1

Among the unknown parameters, we prefix the parameter $\delta$, the inverse of the average time for a person from being exposed to hospitalized, to be 0.14 universally in the algorithm. According to^44^, the mean duration of incubation period is 5.2 days. Furthermore, we assume that the average time for an individual from showing symptoms to being hospitalized is 2 days^45,46^. Thus, the mean duration for an individual from being exposed to being hospitalized is 7.2 days, whose inverse value is approximately 0.14.

#### Step 1: Estimate $\{\gamma _k\}_{k=1}^n$

We first estimate $\{\gamma _k\}_{k=1}^n$ by maximizing the likelihood

$$\begin{aligned} {P\left( \left\{ ( R_a)_k(i), ( C_a)_k(i), k=1,\dots ,n\right\} _{i=1}^T \bigg |\{\gamma _k\}_{k=1}^n \right) } \end{aligned}$$

over $\gamma _k$ for each *k*. We assume that the newly removed cases in one day follow a Poisson distribution whose mean equals to the product of $\gamma _k$ and the accumulated hospitalized cases (which is the difference between the accumulated confirmed cases and the accumulated removed cases, and thus is observable) the day before. Then, the likelihood of $\{\gamma _k\}_{k=1}^n$ can be written as

$$\begin{aligned} &P\left( \left\{ ( R_a)_k(i), ( C_a)_k(i), k=1,\dots ,n\right\} _{i=1}^T \bigg |\{\gamma _k\}_{k=1}^n \right) \\& \quad = \prod _{i=1}^{T} P\left( \left\{ (\Delta R_a)_k(i), k=1,\dots ,n\right\} \bigg |\{\gamma _k\}_{k=1}^n, \left\{ ( R_a)_k(i-1), ( C_a)_k(i-1), k=1,\dots ,n\right\} \right) \\& \quad = \prod _{i=1}^{T}\prod _{k=1}^{n}\text {Pois} \left( (\Delta R_a)_k(i) \big |\gamma _k\left( (C_a)_k(i-1)-(R_a)_k(i-1) \right) \right) \\& \quad = \prod _{k=1}^{n}\prod _{i=1}^{T} \frac{ \left( \gamma _k\left( (C_a)_k(i-1)-(R_a)_k(i-1) \right) \right) ^{ (\Delta R_a)_k(i) }}{ \left( (\Delta R_a)_k(i)\right) ! }\exp \left( - \gamma _k\left( (C_a)_k(i-1)-(R_a)_k(i-1) \right) \right) , \end{aligned}$$

where $\mathrm {Pois}(k\big |\beta )$ ($k\in \mathbb {N}, \beta >0$) denotes the probability that *k* occurrences are observed for a discrete random variable *X* having a Poisson distribution with mean $\beta$.

Then, we estimate $\gamma _k^* = \arg \max _{\gamma _k} \prod _{i=1}^T \text {Pois}\left( (\Delta R_a)_k(i) \big | ((C_a)_k(i-1) - (R_a)_k(i-1))\gamma _k\right)$ for each *k* separately.

#### Step 2: Estimate ${\overline{\Theta }} = \{E_k(0), H_k(0), \lambda _k\}_{k=1}^n$

Next, we estimate the remaining parameters ${\overline{\Theta }} = \{E_k(0), H_k(0), \lambda _k\}_{k=1}^n$, by finding ${\overline{\Theta }}$ that achieves maximum a posteriori probability (MAP).

**Posterior distribution of** ${\overline{\Theta }}$ **and MAE estimate.** We denote the posterior distribution of ${\overline{\Theta }}$ given data $\{(\Delta C_a)_k(i),k=1,\dots ,n\}_{i=1}^T$ as $\pi ({\overline{\Theta }})$. Then by Bayesian formula,

2.5 $$\begin{aligned} \pi ({\overline{\Theta }})=P\left( {\overline{\Theta }} \bigg | \left\{ (\Delta C_a)_k(i)\right\} _{k,i}\right) = \dfrac{1}{Z}P\left( \left\{ (\Delta C_a)_k(i)\right\} _{k,i}\bigg |{\overline{\Theta }}\right) P({\overline{\Theta }}), \end{aligned}$$

where $P({\overline{\Theta }})$ is the prior distribution of ${\overline{\Theta }}$ to be determined and $Z = P\left( \left\{ (\Delta C_a)_k(i)\right\} _{k,i}\right)$ is a constant irrelevant to ${\overline{\Theta }}$. We further denote

2.6 $$\begin{aligned} V({\overline{\Theta }}) =-\log \left( P\left( \left\{ (\Delta C_a)_k(i)\right\} _{k,i} \bigg | {\overline{\Theta }}\right) \right) - \log (P({\overline{\Theta }})), \end{aligned}$$

then

2.7 $$\begin{aligned} \pi ({\overline{\Theta }})=\dfrac{1}{Z}\exp (-V({\overline{\Theta }})). \end{aligned}$$

To fit the realistic evolution of the epidemic more precisely, ${\overline{\Theta }}$ is estimated as

2.8 $$\begin{aligned} {\overline{\Theta }}^* = \arg \max \pi ({\overline{\Theta }}) = \arg \min V({\overline{\Theta }}), \end{aligned}$$

with reasonable prior distribution $P({\overline{\Theta }})$. Then, MCMC sampling scheme starting from ${\overline{\Theta }}^*$ is applied to get the posterior distribution for ${\overline{\Theta }}$. This process might possess higher computational efficiency than choosing the initial point for MCMC randomly or empirically.

Next, we specify the formulas for the likelihood function $P\left( \left\{ (\Delta C_a)_k(i)\right\} _{k,i}\bigg |{\overline{\Theta }}\right)$ and the prior distribution $P({\overline{\Theta }})$.

**Likelihood function of** ${\overline{\Theta }}$. Notice that the ODE system (2.4) is the mean-field version of our stochastic model, and $\{(\widetilde{\Delta C_a})_k(i)\}_{k,i}$ are determined by the parameters ${\overline{\Theta }}$ ($\delta$ and $\{\gamma \}_{k=1}^n$ are treated as given), thus by the Markov property, $\{(\Delta C_a)_k(i)\}$ are all independent for $k=1,\dots , n$, $i=1,\dots ,T$ conditioned on the parameters ${\overline{\Theta }}$. Furthermore we suppose that $(\Delta C_a)_k(i) \sim \text {Pois}((\widetilde{\Delta C_a})_k(i))$. Thus, the likelihood of ${\overline{\Theta }}$ can be written as

2.9 $$\begin{aligned} P\left( \left\{ (\Delta C_a)_k(i)\right\} _{k,i} | {\overline{\Theta }}\right) = \prod _{i=1}^{T} P\left( \left\{ (\Delta C_a)_k(i)\right\} _{k=1}^n \bigg |{\overline{\Theta }}\right) = \prod _{i=1}^{T}\prod _{k=1}^{n}\dfrac{((\widetilde{\Delta C_a})_k(i))^{(\Delta C_a)_k(i)}}{((\Delta C_a)_k(i))!}e^{-(\widetilde{\Delta C_a})_k(i)}. \end{aligned}$$

**Choice of prior distribution of** ${\overline{\Theta }}$. The remaining problem is to choose the prior distribution $p({\overline{\Theta }})$. Presuming that the transmission rates in the regions owning more similarities are closer, $p({\overline{\Theta }})$ is designed to combine the information of correlations between regions. In particular, given a matrix *A* which characterizes the pairwise similarities between the regions, and if we denote ${\lambda } = (\lambda _{1},\dots ,\lambda _{n})^T\in \mathbb {R}^n$,

2.10 $$\begin{aligned} P({\overline{\Theta }}) = \frac{1}{C_{\sigma ,A}} \exp (-{\lambda }^T (D-A){\lambda } - \sigma \Vert {\lambda }\Vert _2^2), \end{aligned}$$

where $D = \text {diag}\{d_1,\dots ,d_n\}$ is the degree matrix of *A* with $d_i = \sum _{j=1}^i A_{ij}$, $C_{\sigma ,A} = \int _{\mathbb {R}^{n}} \exp (-{\lambda }^T (D-A){\lambda } - \sigma \Vert {\lambda }\Vert _2^2) d{\lambda }$ is a constant depending on $\sigma$ and *A*. Here, a small $\sigma$ is chosen for $p({\overline{\Theta }})$ to be a probability measure without imposing much restriction on $\lambda$. Then, by (2.6) and (2.10)

2.11 $$\begin{aligned} V({\overline{\Theta }}) =&-\log \left( P\left( \left\{ (\Delta C_a)_k(i)\right\} _{k,i} \bigg | {\overline{\Theta }}\right) \right) + {\lambda }^T (D-A){\lambda } +\sigma \Vert {\lambda }\Vert _2^2 + \log {C_{\sigma ,A}} \nonumber \\ =&-\log \left( P\left( \left\{ (\Delta C_a)_k(i)\right\} _{k,i} \bigg | {\overline{\Theta }}\right) \right) + \frac{1}{2}\sum _{i,j} a_{ij} (\lambda _{i}-\lambda _{j})^2 + \sigma \sum _i \lambda _i^2 + \log {C_{\sigma ,A}}. \end{aligned}$$

The parameter estimation procedure could be extended to the case when $\{\lambda _k\}$ are time-varying by modifying (2.13), which we will introduce in more detail in “Model extension by allowing time-varying parameters” for the real-world data in China and Europe.

** Construction of affinity matrix** *A* **appearing in prior distribution** (2.10). Now, we specify the construction of affinity matrix *A* in (2.10) that reflects the similarity between regions. *A* is constructed from affinity matrix *W* by further addressing the correlation between regions with more similarities. To treat different data sets and *W* with a unified approach, we assume that $\max _{i,j}W_{ij}=1$ (*W* can be re-scaled entry-wise if necessary).

For a given $W=(W_{ij})_{i,j\in \{1,\dots ,n\}}$ whose choice is detailed later, the next step of attaining *A* is to divide the *n* regions into *d* groups ($D_1,...,D_d$, $\cup _{m=1}^d D_m = \{1,\dots ,n\}$, and $\forall i\ne j$, $D_i\cap D_j = \emptyset$ ) where the regions in the same groups have more similarities. Then, for given $\beta \in (0,1)$ and a given penalty factor $\mu >0$, $a_{ij}$ is constructed as follows:

2.12 $$\begin{aligned} {a_{ij} = \mu {\left\{ \begin{array}{ll} &{}W_{ij}, \quad \text {if regions } i \text { and } j\text { are in the same group},\\ &{}\beta W_{ij},\text { otherwise}. \end{array}\right. }} \end{aligned}$$

We remark that $\beta$ in (2.12) is taken to be 0.1 for all the experiments in this paper. By constructing *A* as in (2.12), correlations for regions in the same groups are further addressed, whose transmission parameters are imposed with stronger restrictions.

Now we specify the choice of *W* for data sets that will be analyzed later in this paper. For simulated data and real-world data in China, in which cases the transportation data are available, we construct *W* from the traveling volume matrices $\{W_i\}_{i=1}^T$. Specifically, $W{:=}{\bar{W}}/\max _{i,j}{\bar{W}}_{ij}$, where ${\bar{W}} := \frac{1}{2} ( (\frac{1}{T}\sum _{l=1}^TW_l) + (\frac{1}{T}\sum _{l=1}^TW_l)^T)$. Nevertheless, for real-world data in Europe, where we are not aware of traveling data publicly available that are sufficient for the proposed model, *W* is just the all 1 adjacency matrix. We remark that the affinity matrix *W* may also be obtained by ways other than using the transportation data, as long as it reflects the similarities between districts.

**Specified formula for MAP estimate of** ${\overline{\Theta }}$. From the MAP estimate (2.8), definition of *V* (2.11) given prior distribution (2.10), and the definition of $a_{ij}$ in (2.12), the inference of ${\overline{\Theta }}$ can be equivalently written as follows

2.13 $$\begin{aligned} { {\overline{\Theta }}^*}&{= \arg \min V({\overline{\Theta }})} \nonumber \\&{= \arg \min \left( -\log \left( P\left( \left\{ (\Delta C_a)_k(i)\right\} _{k,i} \bigg | {\overline{\Theta }}\right) \right) + \frac{1}{2}\sum _{i,j} a_{ij} (\lambda _{i}-\lambda _{j})^2 + \sigma \sum _i \lambda _i^2\right) } \nonumber \\&= \arg \min \left( -\log \left( P\left( \left\{ (\Delta C_a)_k(i)\right\} _{k,i} \bigg | {\overline{\Theta }}\right) \right) + \frac{\mu }{2}\left( \sum _m\sum _{i,j\in D_m} W_{ij} (\lambda _{i}-\lambda _{j})^2 \right. \right. \nonumber \\&\quad \left. \left. + \beta \sum _{m_1<m_2}\sum _{i\in D_{m_1},j\in D_{m_2}} W_{ij} (\lambda _{i}-\lambda _{j})^2\right) + \sigma \sum _i \lambda _i^2 \right) , \end{aligned}$$

where $\left( P\left( \left\{ (\Delta C_a)_k(i)\right\} _{k,i} \bigg | {\overline{\Theta }}\right) \right)$ is given in (2.9).

It can be seen that by choosing the prior distribution as in (2.10), a $l_2$ regularization term is imposed for better generalization.

**Estimation of the marginal posterior distribution of** ${\overline{\Theta }}$. Finally, after choosing $P({\overline{\Theta }})$ determined by *A* and $\sigma$, the optimization process ${\overline{\Theta }}^* = \arg \min V({\overline{\Theta }})$ as in (2.13) is accomplished by a BFGS algorithm^47–50^. To obtain the posterior distribution of ${\overline{\Theta }}$, we use classical MCMC sampling scheme starting from ${\overline{\Theta }}^*$ solved by the optimization.
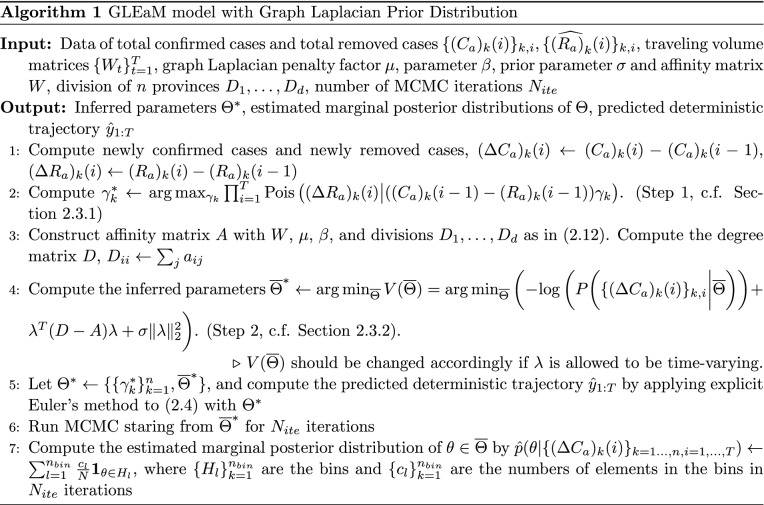


### Prediction of the epidemic trajectories with the estimated parameters

Once the parameters $\Theta ^*=\{\{\gamma _k^*\}_{k=1}^n, {\overline{\Theta }}^*\}$ are estimated from the optimizations $\gamma _k^* = \arg \max _{\gamma _k} \prod _{i=1}^T \text {Pois}\bigg ((\Delta R_a)_k(i) \big | ((C_a)_k(i-1) - (R_a)_k(i-1))\gamma _k\bigg )$ and ${\overline{\Theta }}^* = \arg \min V({\overline{\Theta }})$ as described in “Estimation of model parameters”, the trajectories of newly confirmed cases could be simulated according to the stochastic dynamic process with $\Theta ^*$. Furthermore, trajectories could also be sampled from the posterior distribution of $\Theta$ instead of using $\Theta ^*$ alone, which also takes the randomness from ${\overline{\Theta }}$ into account. Particularly, this could be achieved by sampling ${\overline{\Theta }}$ from MCMC and then simulating trajectories with the sampled $\{\{\gamma _k^*\}_{k=1}^n, {\overline{\Theta }}\}$. Additionally, deterministic trajectories determined by (2.4) could also be computed by explicit Euler’s method.

## Experimental results for simulated data

Two specific cases are considered for simulated data. We first remark that the regions in “Methods” are called as provinces in this section. Section “Four provinces case” considers four provinces separated into two groups (the provinces in the same group are assumed to have more similarities) and with traffic between each pair of the provinces. Section “Thirty provinces case” considers thirty provinces randomly separated into three groups, with the other settings similar to the previous case. Section “More details of experimental settings and sensitivity analysis” includes more details of the experimental settings and sensitivity analysis.

### More details of experimental settings and sensitivity analysis

#### Experimental settings

The results in “Experimental results for simulated data” are for 100 replicas. In each replica, three random trajectories are sampled independently according to the stochastic model with prefixed parameters, part of which are treated as the ground truth training, validation, and testing trajectory, respectively (see more details in Sect. A.1.1 of Supplementary Information).

For each model, we first fit the parameters using the training trajectory and then predict the testing trajectory using the estimated parameters. Note that we detail the choice of hyper-parameters for the model proposed in Sect. A.2 of Supplementary Information. In particular, the penalty factor $\mu >0$ is chosen by cross-validation and chosen as the value minimizing the validation error, since for the simulated data, the validation error are identically distributed as the testing error. The comparison of trajectory prediction is from one typical realization, for which we compare the ground truth training and testing trajectories with the fitted training and predicted testing trajectories for all the models. Additionally, parameter estimation and quantitative evaluations are compared with mean and standard deviation over all 100 replicas. The detailed computations of training, validation, and testing errors can be found in Sect. B of Supplementary Information.

#### Sensitivity analysis of $\sigma$

Note that parameter inference with the proposed model involves the parameter $\sigma$, as shown in (2.13). Therefore, the sensitivity analysis for the parameter $\sigma$ in (2.13) is performed for the four provinces case. Specifically, the results for $\sigma$ varying from $10^{-6}$ to $10^0$ are presented and compared. Details can be found in “Results of parameter estimation” and “Further model evaluation”. Similar results are obtained for other data sets, and details are omitted.

#### Mismatched partition of regions

We also note that the graph Laplacian penalty of the proposed model depends on the partitioning the regions into several groups, as described in “Estimation of model parameters”. Since the graph knowledge is usually not fully known, it is a question whether our methods can still perform well without accurate prior knowledge. For the thirty provinces case, we report the results of the proposed model with a mismatch between the partition of the regions and the ground truth division, the details of which can be found in “Thirty provinces case”.

### Four provinces case

#### Data description

In this simulated study, we let $n=4$, $T=20$, and set the threshold $T_{th}$ separating training and testing data to be 10 (more detailed can be seen in Supplementary Information S1). The other prefixed parameters are listed below:

- For $$k\in \{1,2,3,4\}$$, $$N_k(0) = 10^6$$, $$E_k(0)=30$$, $$H_k(0)=10$$.
- For $$l\in \{1,\dots ,T\}$$, $$i,j\in \{1,2,3,4\}$$ and $$i\ne j$$, $$(W_l)_{ij} = 5\times 10^3$$.
- $$\lambda _1=0.5$$, $$\lambda _2=0.47$$, $$\lambda _3=0.4$$, $$\lambda _4=0.37$$, $$\delta =\gamma _1=\cdots =\gamma _4=0.14$$.

The four provinces are divided into two groups, with the first group consisting of Provinces 1 and 2 and the second group consisting of Provinces 3 and 4. The similarities within groups are reflected in the settings that the values of $\{\lambda _k\}$ are closer for provinces in the same group.

#### Models to compare

**The proposed model and other four baseline models.** We first specify the models to be compared below. The last one is the proposed model, and the first four models serve as baselines with different settings.

1. The model with uniform prior distribution, without heterogeneity or migration.
2. The model with uniform prior distribution, without heterogeneity but with migration.
3. The model with uniform prior distribution, with heterogeneity but without migration.
4. The model with uniform prior distribution, with both heterogeneity and migration.
5. The model with prior distribution based on graph Laplacian, with both heterogeneity and migration.

For better illustration and comparison between the models in the experiment results, the Models 1–5 are summarized in Table 2 below.

**Table 2:** Models to be compared when the transportation data are available.

Model	Migration	Heterogeneity	Prior of $\Theta$
1	$\times$	$\times$	Uniform prior
2	$\checkmark$	$\times$	Uniform prior
3	$\times$	$\checkmark$	Uniform prior
4	$\checkmark$	$\checkmark$	Uniform prior
5	$\checkmark$	$\checkmark$	Graph Laplacian prior

Model 5 is the proposed model in this paper, and Models 1–4 are baseline models with different settings.

First, the models with uniform prior distributions themselves (Models 1–4) are compared according to whether two key assumptions exist in the model:

1. Whether the transmission rates $\{\lambda _k\}$ are allowed to vary over regions.
2. Whether there exists transportation between regions.

Then, the model with prior distribution based on graph Laplacian (Model 5) is compared with those using uniform distributions as prior distributions (Models 1–4). The former one utilizes the correlation between subpopulations by adding a $l_2$ regularization term for the model. In contrast, only lower and upper bounds are imposed on parameters without other prior information being used in the latter ones.

**Parameter inference of the five models and sensitivity of** $\sigma$. For Model 5, the proposed model, the parameters $\Theta = \{\{\gamma _k\}_{k=1}^n, {\overline{\Theta }}\}$ are estimated following the two-step procedure described in “Estimation of model parameters”, where ${\overline{\Theta }} = \{E_k(0), H_k(0), \lambda _k\}_{k=1}^n$. For the estimation of ${\overline{\Theta }}$, following the general formula (2.13) in “Estimation of model parameters”, the specific formula of ${\overline{\Theta }}^*$ for the four provinces case is as follows,

3.1 $$\begin{aligned} {\overline{\Theta }}^* =\arg \min (-\log p(y_{1:T_{th}}|{\overline{\Theta }}) + \mu \left( (\lambda _1-\lambda _2)^2 + (\lambda _3-\lambda _4)^2 + \beta \sum _{(i,j)\ne (1,2) \text {or} (3,4)}(\lambda _i-\lambda _j)^2\right) + \sigma \Vert \lambda \Vert _2^2 ), \end{aligned}$$

where $\beta$ is taken to be 0.1. For Model 5, we conduct the sensitivity analysis for parameter $\sigma$ in (3.1) and present results for $\sigma =10^{-6},10^{-3}$ and $10^0$ respectively in “Results of parameter estimation” and “Further model evaluation”.

We remark that in Models 1–4, the estimation of $\Theta =\{\{\gamma _k\}_{k=1}^n, {\overline{\Theta }}\}$ still follows a similar two-step procedure as in Model 5, and the first step of obtaining $\gamma _k^* = \arg \max _{\gamma _k} \prod _{i=1}^T \text {Pois}\big ((\Delta R_a)_k(i) \big | ((C_a)_k(i-1) - (R_a)_k(i-1))\gamma _k\big )$ remains formally the same. The difference lies in the optimization object of ${\overline{\Theta }}$. First, the $l_2$ regularization term becomes prior knowledge of the parameters’ upper and lower bounds. Second, for models without heterogeneity of parameters, $\{\lambda _k\}_{k=1}^n$ are forced to be the same in ODE system (2.4). For models without transportation between regions, terms involving $W_t$ disappear in (2.4). Additionally, for the other data sets considered in the following sections, the parameter estimation methods for the baseline models are similar and thus will not be repeated.

Finally, note that the model in Ref.^12^ is similar to Models 1 and 2, since they all assume a spatially homogeneous transmission parameter. However, Ref.^12^ assumed that the epidemic was seeded from one seed region while Models 1 and 2 do not make such assumption. Moreover, Ref.^12^ focused more on the spread of the epidemic from the seed region at the early stage of the pandemic, and only the introduction dates in the other regions were utilized for the estimation of transmission parameters. In comparison, the estimation of transmission rate in Models 1 and 2 exploits the data in all regions in the whole process.

#### Results of trajectory prediction

First, we remark that in Model 5, $\mu$ is chosen to be the minimizer of the averaged validation errors over 100 replicas over a range of values of $\mu$. The weighted (simply averaged) validation errors, MAE$^{[\mathrm Val]}_{(w)}$ and MSE$^{[\mathrm Val]}_{(w)}$ (MAE$^{[\mathrm Val]}_{(s)}$ and MSE$^{[\mathrm Val]}_{(s)}$), are defined as in Sect. B of Supplementary Information. We remark that the superscript $^{[\mathrm Val]}$ refers to when the error is computed on validation data, and the subscripts $_{(w)}$ and $_{(s)}$ denote that the errors are the weighted and simple average of relative errors over time respectively.

The averaged weighted validation errors MAE$^{[\mathrm Val]}_{(w)}$ and MSE$^{[\mathrm Val]}_{(w)}$ over replicas are shown in Supplementary Fig. S2, and the simply averaged counterparts are shown in Supplementary Fig. S3. For parameter inference using Model 5, $\mu$ is chosen to be $10^{2.7}$, at which all the averaged validation errors (MAE$^{[\mathrm Val]}_{(w)}$, MSE$^{[\mathrm Val]}_{(w)}$, MAE$^{[\mathrm Val]}_{(s)}$ and MSE$^{[\mathrm Val]}_{(s)}$) over 100 replicas are minimized, as can be seen from Supplementary Figs. S2 and S3.

**Figure 2:**
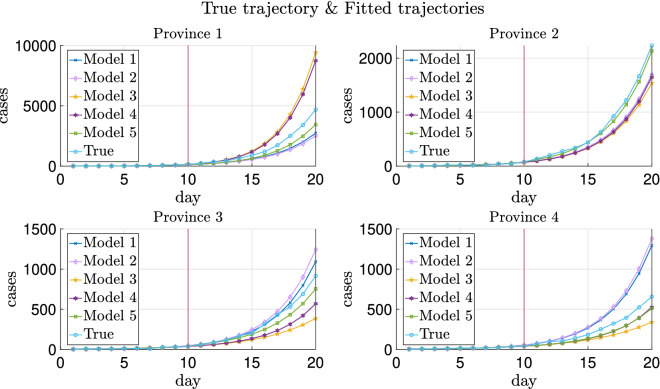
True and fitted trajectories for the simulated data with four provinces. The vertical lines show the threshold of training-testing split. In Model 5, $\mu =10^{2.7}$ is chosen, at which the averaged validation errors over 100 replicas are minimized as shown in Supplementary Figs. S2 and S3.

**Figure 3:**
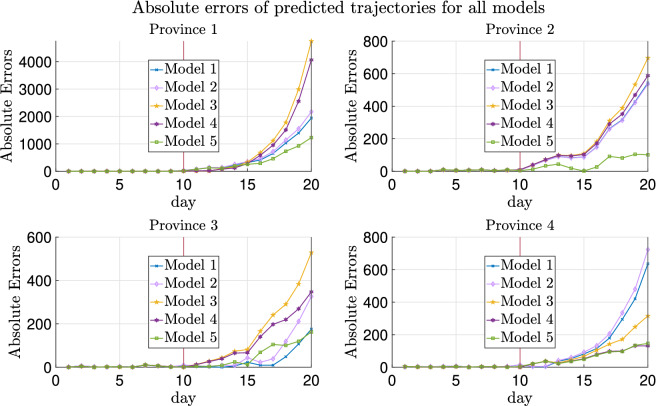
Absolute errors of fitted trajectories of Models 1–5 for the simulated data with four provinces. The vertical lines show the threshold of training-testing split.

The trajectories of a typical realization are plotted in Fig. 2 and the absolute errors of the fitted trajectories are shown in Fig. 3. As can be seen in these two figures, heterogeneity helps improve the prediction of testing data more than transportation, while introducing migration without heterogeneity of parameters worsens the estimate as can also be noticed from Table 4. More explanations can be found in “Further model evaluation”.

Additionally, Model 5 with prior distrbution based on graph Laplacian lowers the absolute errors of predicted trajectories compared with Model 4.

As shown in Figs. 2 and 3, Models 1 and 2 have slightly better generalization accuracy than Model 5 for Province 3. On the one hand, for data in this replica, the estimated $\lambda _3$ is 0.3860 using Model 5 and 0.4640 using Models 1 or 2 (recalling that the ground truth $\lambda _3$ is 0.4). On the other hand, due to the randomness of the generated testing data, the sampled newly confirmed cases are much more than the deterministic ones in Province 3 obtained by running (2.4) with the ground truth parameters. Hence, although all these estimates of $\lambda _3$ are biased from the ground truth 0.4, estimates using Models 1 and 2, which are biased up, lead to less absolute errors.

#### Results of parameter estimation

The mean and standard deviation of $\{\lambda _i\}$ estimated by the five models for four provinces case are reported in Table 3. It can be observed that models allowing heterogeneity estimate parameters more accurately, and Model 5 that integrates the correlation leads to slightly better estimate for $\lambda _2$. We can see that compared to Model 4, the estimates of smaller $\lambda _k$’s (such as $\lambda _2,\lambda _3,\lambda _4$) become larger, and the estimate of $\lambda _1$ which has the largest value becomes smaller, since the graph Laplacian penalty tends to make $\{\lambda _k\}_{k=1}^n$ closer to each other.

Moreover, we performed a sensitivity analysis for the hyper-parameter $\sigma$ to check that the results are robust to $\sigma$. The last two rows of Table 3 show the parameter estimation results for Model 5 with $\sigma =10^{-3}$ and $10^0$ respectively (more values of $\sigma \in [10^{-8}, 10^0]$ are tested and the results are similar as well). We observe that the variation of parameters estimated by Model 5 with $\sigma$ varying from $10^{-6}$ to $10^0$ does not exceed $1\%$. Therefore, the parameter estimation results are not sensitive to the choice of $\sigma$ as long as $\sigma$ is not too large.

**Table 3:** Estimated $\lambda _i$ with standard deviation using Models 1–5 for simulated data with four provinces.

Model	M	H	GL prior	$\lambda _1$	$\lambda _2$	$\lambda _3$	$\lambda _4$
1	$\times$	$\times$	$\times$	$0.4458\pm 0.0192$			
2	$\checkmark$	$\times$	$\times$	$0.4459\pm 0.0192$			
3	$\times$	$\checkmark$	$\times$	$0.4927\pm 0.0331$	$0.4620\pm 0.0370$	$0.3988\pm 0.0389$	$0.3705\pm 0.0349$
4	$\checkmark$	$\checkmark$	$\times$	$0.4980\pm 0.0337$	$0.4643\pm 0.0376$	$0.3922\pm 0.0407$	$0.3605\pm 0.0366$
5	$\checkmark$	$\checkmark$	$\checkmark (\sigma =10^{-6})$	$0.4805\pm 0.0254$	$0.4654\pm 0.0260$	$0.3979\pm 0.0256$	$0.3879\pm 0.0231$
	$\checkmark$	$\checkmark$	$\checkmark (\sigma =10^{-3})$	$0.4805\pm 0.0254$	$0.4654\pm 0.0260$	$0.3979\pm 0.0256$	$0.3879\pm 0.0231$
	$\checkmark$	$\checkmark$	$\checkmark (\sigma =10^{0})$	$0.4800\pm 0.0254$	$0.4648\pm 0.0260$	$0.3972\pm 0.0256$	$0.3871\pm 0.0230$

Recall that the ground truth is $\lambda _1=0.5$, $\lambda _2=0.47$, $\lambda _3=0.4$, $\lambda _4=0.37$. Models in Column 1 are detailed in Table 2. Columns 2–4 indicate whether the model permits migration between provinces (for “M”), the heterogeneity of parameters (for “H”) and the Graph Laplacian prior (for “GL prior”), respectively. $\lambda _i$ are inferred for each of the 100 replicas, and then the mean and standard deviation are presented in the table above. In addition, for parameter inference using Model 5 $\mu =10^{2.7}$, ($\sigma =10^{-6},10^{-3},10^0$ respectively), at which the averaged relative validation errors over 100 replicas are minimized as shown in Supplementary Figs. S2 and S3.

#### Further model evaluation

The training and testing errors, MAE$^{[\mathrm Tr]}_{(w)}$, MAE$^{[\mathrm Te]}_{(w)}$, MSE$^{[\mathrm Tr]}_{(w)}$, MSE$^{[\mathrm Te]}_{(w)}$, as defined in Sect. B (in the Eq. (S5)) of Supplementary Information, are listed below in Table 4 with mean and standard deviation. We remind the readers that the superscripts $^{[\mathrm Tr]}$ and $^{[\mathrm Te]}$ represent the errors are computed on training and testing data respectively, and the subscript $_{(w)}$ denotes that the error is weighted average of the daily relative errors over time. It can be seen from Table 4 that the presence of both heterogeneity and transportation helps reduce the training and testing errors by comparing the first four models. By comparing Model 4 and Model 5, it can be seen that using the graph Laplacian regularization leads to better prediction performance in average, which might not be obvious in this case due to the relatively large variance. The advantage of the proposed Model 5 is more evident for larger number of regions involved in the dynamic system, as shown in the next “Thirty provinces case”.

**Table 4:** Training and testing errors with standard deviation of Models 1–5 for simulated data with four provinces.

Model	M	H	GL prior	MAE$^{[\mathrm Tr]}_{(w)}$	MAE$^{[\mathrm Te]}_{(w)}$	MSE$^{[\mathrm Tr]}_{(w)}$	MSE$^{[\mathrm Te]}_{(w)}$
1	$\times$	$\times$	$\times$	$0.1728\pm 0.0273$	$0.4700\pm 0.1879$	$0.2379\pm 0.0375$	$0.4801\pm 0.1887$
2	$\checkmark$	$\times$	$\times$	$0.1828\pm 0.0296$	$0.5432\pm 0.2048$	$0.2487\pm 0.0390$	$0.5551\pm 0.2067$
3	$\times$	$\checkmark$	$\times$	$0.1417\pm 0.0232$	$0.4095\pm 0.1692$	$0.1997\pm 0.0348$	$0.4173\pm 0.1683$
4	$\checkmark$	$\checkmark$	$\times$	$0.1418\pm 0.0229$	$0.3834\pm 0.1672$	$0.1997\pm 0.0343$	$0.3900\pm 0.1665$
5	$\checkmark$	$\checkmark$	$\checkmark (\sigma =10^{-6})$	$0.1481\pm 0.0230$	$0.3300\pm 0.1519$	$0.2069\pm 0.0355$	$0.3365\pm 0.1513$
	$\checkmark$	$\checkmark$	$\checkmark (\sigma =10^{-3})$	$0.1481\pm 0.0230$	$0.3300\pm 0.1519$	$0.2069\pm 0.0355$	$0.3365\pm 0.1513$
	$\checkmark$	$\checkmark$	$\checkmark (\sigma =10^{0})$	$0.1482\pm 0.0230$	$0.3283\pm 0.1498$	$0.2071\pm 0.0356$	$0.3347\pm 0.1490$

The formulas of errors are detailed in Sect. B (Eq. (S5)) of Supplementary Information. Remarks for Columns 1–4 and the choice of $\mu =10^{2.7}$ in Model 5 are the same as those in Table 3. MAE$^{[\mathrm Tr]}_{(w)}$, MAE$^{[\mathrm Te]}_{(w)}$, MSE$^{[\mathrm Tr]}_{(w)}$ and MSE$^{[\mathrm Te]}_{(w)}$ are computed for each of the 100 replicas, then the mean and standard deviation are presented in the table above.

In addition, it can be seen from Table 4 that the errors increase greatly after transportation is included while heterogeneity remains absent. A possible explanation for this might be that without heterogeneity of parameters and transportation between provinces, the estimated values of $\lambda _k$’s are lower than the true values of $\lambda _k$’s for group 1, which leads to that the estimated newly confirmed cases are fewer than the true ones for provinces in group 1. For the same reason, the estimated newly confirmed cases are higher than the true ones in group 2. When the transportation is considered, more confirmed cases in group 1 are transferred to group 2 than the cases transported in the opposite direction. As a result, when the transmission parameters do not have heterogeneity, migration between provinces will worsen the prediction performance compared to the case without migration.

Furthermore, the last two rows of Table 4 report the training and testing errors for Model 5 with the same $\mu =10^{2.7}$ while $\sigma =10^{-3}$ and $\sigma =10^0$ respectively. As a consequence of the robustness of the parameter estimation regarding $\sigma$, the errors of Model 5 are also robust to $\sigma$. The similar analysis is also performed for the other data sets and the similar results can be obtained which we do not report repetitively. Hereinafter, the results are presented with $\sigma =10^{-6}$.

The plots of the mean of weighted and simply averaged testing errors MAE$^{[\mathrm Te]}_{(w)}$, MSE$^{[\mathrm Te]}_{(w)}$, MAE$^{[\mathrm Te]}_{(s)}$ and MSE$^{[\mathrm Te]}_{(s)}$ against varying $\mu$ are shown in Supplementary Figs. S2 and S3 respectively. Recall that the subscripts (*w*) and (*s*) denote the weighted and simple average respectively. Note that Model 5 with $\mu =10^{2.7}$, at which the averaged validation errors over replicas are minimized, achieves the minimal values of testing errors MAE$^{[\mathrm Te]}_{(w)}$ and MSE$^{[\mathrm Te]}_{(w)}$ (also MAE$^{[\mathrm Te]}_{(s)}$ and MSE$^{[\mathrm Te]}_{(s)}$). This is because validation and testing errors have the same distribution in this case.

### Thirty provinces case

In this simulation study, we present the results for the simulated data involving a larger number of regions.

#### Data description

We set the total number of regions $n=30$, the total days considered $T=30$, and set the threshold that separates the training and testing set as $T_{th}=10$. The other prefixed parameters are listed as below:

- For $$k\in \{1,2,\dots ,n\}$$, $$N_k(0) = 10^6$$, $$E_k(0)=30$$, $$H_k(0)=10$$.
- For $$l\in \{1,\dots ,T\}$$, $$i,j\in \{1,2,\dots ,n\}$$ and $$i\ne j$$, $$(W_l)_{ij} = 5\times 10^3$$.
- As in the four provinces case, we assign thirty provinces into different groups and reflect the similarity between provinces in the choice of $\lambda _k$’s, namely the values of $\lambda _k$’s being closer for provinces in the same groups. The three groups are denoted as $D_1, D_2, D_3$ below and the partition is denoted as *P*. Then, the proposed model (Model 5) can be applied as described in “Estimation of model parameters” with or without the graph information (the ground truth partition *P*) being fully known.

**Figure 4:**
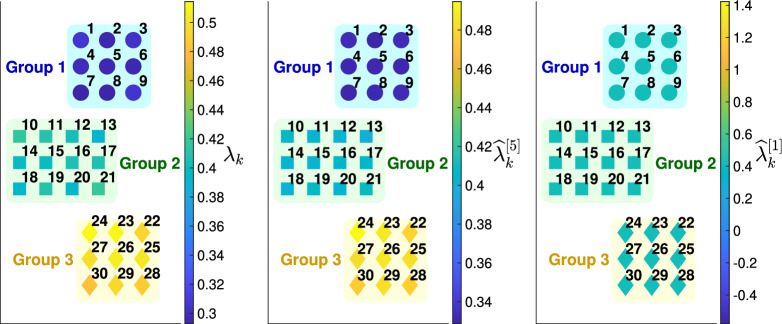
Maps of the thirty provinces divided into three groups, colored by transmission parameters or their estimates. In all the three panels, the circles on top of the panels denote the nine provinces in Group 1 (indexed by 1–9), the squares in the middle of the panels denote twelve provinces in Group 2 (indexed by 10–21), and the diamonds at the bottom of the panels denote nine provinces in Group 3 (indexed by 22–30). The provinces are colored by the ground truth $\{\lambda _k\}_{k=1}^{30}$ in the left panel, by the averaged estimates $\{{\widehat{\lambda }}_k^{[5]}\}_{k=1}^{30}$ using Model 5 in the middle panel, and by the averaged estimates $\{{\widehat{\lambda }}_k^{[1]}\}_{k=1}^{30}$ using Model 1 in the right panel. The superscripts $^{[5]}$ and $^{[1]}$ denote the models used to obtain the estimates of $\lambda _k$. In the experiments, the traveling volumes between provinces are taken to be constant.

Figure 4 shows maps of the thirty provinces colored by transmission parameters or their estimates. It can be observed that $\lambda _k$’s of provinces in the same groups are closer. Furthermore, the estimates of $\lambda _k$’s using Model 5 with heterogeneity are close to the ground truth. In contrast, the estimates using Model 1 without heterogeneity are the same for all the provinces and deviate from the ground truth $\lambda _k$ for most of the provinces. More results of trajectory prediction and parameter estimation can be found in “Results of trajectory prediction” and “Results of parameter estimation”.

Details of assignment of the thirty provinces, choice of transmission rates and their values are listed in Sect. C of Supplementary Information.

#### Models to compare

We compare the Models 1–5 listed in Table 2 as detailed in “Models to compare”.

**Parameter inference of the five models.** For Model 5, the estimation of $\Theta$ still follows the procedure in “Estimation of model parameters”. For the inference of ${\overline{\Theta }}$, when we assume that the ground truth group division *P* is known, the general formula (2.13) can be specified as follows:

3.2 $$\begin{aligned} {\overline{\Theta }}^*&= \arg \min \Bigg (-\log \left( p\left( y_{1:T_{th}}| {\overline{\Theta }}\right) \right) + \frac{\mu }{2}\Bigg (\sum _{m=1}^3\sum _{i,j\in D_m} (\lambda _{i}-\lambda _{j})^2 \nonumber \\&\quad + \beta \sum _{1\le m_1<m_2\le 3}\sum _{i\in D_{m_1},j\in D_{m_2}} (\lambda _{i}-\lambda _{j})^2\Bigg ) + \sigma \sum _i \lambda _i^2 \Bigg ), \end{aligned}$$

where $\beta =0.1, \sigma =10^{-6}$. The parameter inference for Models 1–4 is the same as described in “Models to compare”.

**Mismatched partitions of regions.** For Model 5, we also report results when there are mismatches in partition of the provinces, since in real-world applications the graph information may not be fully known. Specifically, since most of the results reported in the following sections are for Provinces 1 (in $D_1$), 10 (in $D_2$), and 22 (in $D_3$), we consider the other two partitions $P'=\{D_1',D_2',D_3'\}$ and $P''=\{D_1'',D_2'',D_3''\}$, which are different from *P* in the assignments of Provinces 1, 10, and 22, and another three provinces respectively. Comparison between the results using the ground truth partition and the results of these two different kinds of mismatches may reflect the potentially different impact of these mismatches on the results. For Model 5 with partitions $P'$ or $P''$, ${\overline{\Theta }}$ is estimated by (3.2) with $P=\{D_1,D_2,D_3\}$ replaced by $P'=\{D_1',D_2',D_3'\}$ or $P''=\{D_1'',D_2'',D_3''\}$.

The partition $P'=\{D_1',D_2',D_3'\}$ deviates from *P* in the assignment of Provinces 1 (in $D_1$), 10 (in $D_2$), and 22 (in $D_3$):

- Group 1 ($$D_1'$$): Provinces **22**, 2–9.
- Group 2 ($$D_2'$$): Provinces **1**, 11–21.
- Group 3 ($$D_3'$$): Provinces **10**, 23–30.

The partition $P''=\{D_1'',D_2'',D_3''\}$ deviates from *P* in the assignment of Provinces 2 (in $D_1$), 11 (in $D_2$), and 23 (in $D_3$):

- Group 1 ($$D_1''$$): Provinces 1, **23**, 3–9.
- Group 2 ($$D_2''$$): Provinces 10, **2**, 12–21.
- Group 3 ($$D_3''$$): Provinces 22, **11**, 24–30.

For mismatched partitions $P'$ and $P''$, the provinces assigned to the wrong groups are marked in bold.

#### Results of trajectory prediction

Note that for Model 5 (with the ground truth partition *P*), we choose $\mu ^*=10^{1.8}$, at which the averaged validation errors over replicas are minimized, as marked with orange pentagrams in Fig. 7 and Supplementary Fig. S4. The following results for Model 5 are all obtained with $\mu ^*=10^{1.8}$.

**Figure 5:**
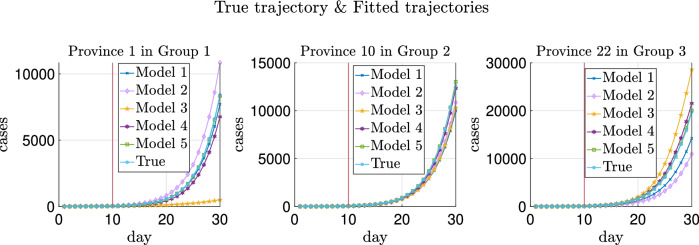
True and fitted trajectories for the simulated data in the three provinces chosen from the total thirty provinces. The vertical lines show the threshold of training-testing split. For Model 5, $\mu ^*=10^{1.8}$, at which the averaged validation errors over replicas are minimized (as shown in Fig. 7).

**Figure 6:**
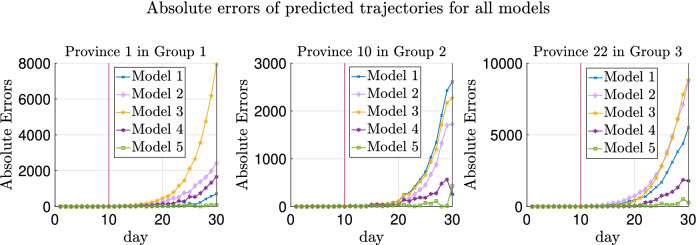
Absolute errors of the fitted trajectories for simulated data in the three provinces chosen from the total thirty ones. The vertical lines show the threshold of training-testing split.

We choose three provinces from the total thirty provinces (one province from each group), and plot the prediction of the trajectories and also the absolute errors from one specific replica as in Figs. 5 and 6. It can be observed from Fig. 6 that Model 5 (the green lines) achieve the most accurate prediction results due to the graph Laplacian regularization. Model 4 (the dark purple lines) also have good generalization performance, thanks to the heterogeneity and transportation involved in this model.

We note that Model 3 also behaves much worse than the best models, especially in Provinces 1 (in $D_1$) and 22 (in $D_3$). In fact, the estimates for the $\lambda _k$’s in these three provinces using Model 3 are 0.2908, 0.4198, and 0.4639, respectively, which are not much different from the ground truth values. Nevertheless, Model 3 does not involve transportation between provinces. This leads to that the confirmed cases of Province 22 in $D_3$ (which are much more than the cases of provinces in the other two groups) are not output to other provinces; hence the predicted newly confirmed cases are much more than the truth. A similar situation happens in the case of Province 1 (in $D_1$). Namely, Model 3 does not take the imported cases from the provinces in the other two groups into account, causing the predicted newly confirmed cases to be much less than the truth.

#### Results of parameter estimation

The mean and standard deviation of $\lambda _k$’s in Provinces 1 (in $D_1$), 10 (in $D_2$), and 22 (in $D_3$) are reported in Table 5. We can still see that Models 3–5 estimate the transmission parameters more accurately than Models 1 and 2 especially for Provinces 1 and 22. In addition, the estimates by Model 5 of $\lambda _k$ are closer than those by Model 4, due to the existence of the regularization term.

**Table 5:** Estimated $\lambda _i$ with standard deviation using Models 1–5 for simulated data with thirty provinces.

Model	M	H	GL prior	$\lambda _1$	$\lambda _{10}$	$\lambda _{22}$
1	$\times$	$\times$	$\times$	$0.4223\pm 0.0067$		
2	$\checkmark$	$\times$	$\times$	$0.4227\pm 0.0067$		
3	$\times$	$\checkmark$	$\times$	$0.3743\pm 0.0349$	$0.4101\pm 0.0284$	$0.4537\pm 0.0324$
4	$\checkmark$	$\checkmark$	$\times$	$0.3051\pm 0.0383$	$0.4093\pm 0.0350$	$0.4902\pm 0.0326$
(*P*)	$\checkmark$	$\checkmark$	$\checkmark$	$0.3332\pm 0.0172$	$0.4071\pm 0.0175$	$0.4784\pm 0.0209$
5($P'$)	$\checkmark$	$\checkmark$	$\checkmark$	$0.3669\pm 0.0148$	$0.4335\pm 0.0180$	$0.4375\pm 0.0218$
($P''$)	$\checkmark$	$\checkmark$	$\checkmark$	$0.3430\pm 0.0174$	$0.4051\pm 0.0174$	$0.4761\pm 0.0210$

**Figure 7:**
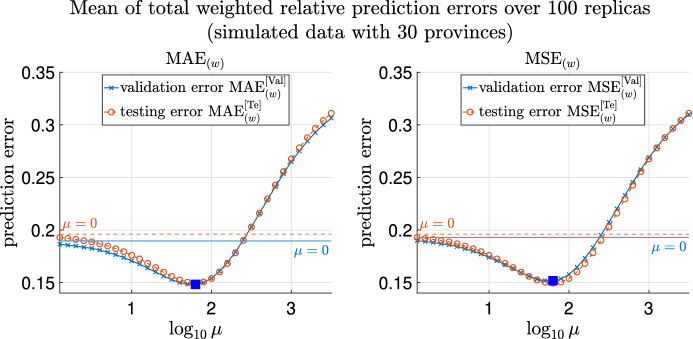
Testing and validation errors on simulated data with thirty provinces. Testing and validation errors on simulated data with four provinces. Left: The weighted prediction errors on validation set (MAE$^{[\mathrm Val]}_{(w)}$) and testing set (MAE$^{[\mathrm Te]}_{(w)}$) respectively, plotted vs. the values of $\mu$. The errors are averaged over 100 replicas of experiment. Right: Same plot of MSE error. In each plot, the blue and red horizontal lines show the values of the averaged errors when $\mu = 0$. Note that both the MAE and MSE validation errors are minimized at $\mu ^*=10^{1.8}$, which is marked by blue squares in both plots. The construction of training/validation/testing data is detailed in Sect. A.1.1 of Supplementary Information, and the formulas of computing the errors can be found in Sect. B of Supplementary Information.

The ground truth is $\lambda _1=0.307$ (in $D_1$), $\lambda _{10}=0.416$ (in $D_2$), $\lambda _{22}=0.493$ (in $D_3$) as listed in Sect. C of Supplementary Information. Remarks for Columns 1–4 and estimates of $\lambda _k$’s are the same as those in Table 3. In addition, for inference of parameters using Model 5, $\mu ^*=10^{1.8}$, at which the averaged validation errors over 100 replicas (computed with partition *P*) are minimized as shown in Fig. 7 and Supplementary Fig. S4. The partitions of Model 5 are *P*, $P'$, and $P''$ respectively. *P* is the ground truth underlying graph structure, and $P'$ and $P''$ are mismatched partitions introduced in “Models to compare”.

The last three rows show the parameter estimates using Model 5 with three partitions *P*, $P'$, and $P''$ respectively. Recall that *P* is the ground truth underlying graph structure, and $P'$ and $P''$ are mismatched partitions introduced in “Models to compare”. It can be seen that compared with the estimated parameters using Model 5 with $P'$ that differs from *P* in the grouping of Provinces 1, 10, and 22, the estimates using Model 5 with $P''$ that differs from *P* in the grouping of Provinces 2, 11, and 23 are closer to the estimates using Model 5 with the ground truth partition *P*. Hence, the results imply that for the parameter inference of simulated data generated from a certain partition, the incorrect division causes more discrepancy in the mismatched regions when comparing the estimates using the ground truth partition.

#### Further model evaluation

The mean and standard deviation of the weighted training and testing errors (formulas can be found in Sect. B of Supplementary Information, specifically in Eq. (S5)) are presented in Table 6. We see that Model 5 with $\mu ^*=10^{1.8}$ achieves the minimum testing errors among all the models, and hence have the best generalization performance.

**Table 6:** Training and testing errors of Models 1–5 with standard deviation for simulated data with thirty provinces.

Model	M	H	GL prior	MAE$^{[\mathrm Tr]}_{(w)}$	MAE$^{[\mathrm Te]}_{(w)}$	MSE$^{[\mathrm Tr]}_{(w)}$	MSE$^{[\mathrm Te]}_{(w)}$
1	$\times$	$\times$	$\times$	$0.1739\pm 0.0092$	$0.1704\pm 0.0543$	$0.2445\pm 0.0135$	$0.1744\pm 0.0534$
2	$\checkmark$	$\times$	$\times$	$0.2427\pm 0.0136$	$0.3387\pm 0.0573$	$0.3128\pm 0.0171$	$0.3420\pm 0.0566$
3	$\times$	$\checkmark$	$\times$	$0.1541\pm 0.0082$	$0.4920\pm 0.0594$	$0.2212\pm 0.0137$	$0.4973\pm 0.0599$
4	$\checkmark$	$\checkmark$	$\times$	$0.1549\pm 0.0083$	$0.1960\pm 0.0647$	$0.2200\pm 0.0139$	$0.1994\pm 0.0649$
(*P*)	$\checkmark$	$\checkmark$	$\checkmark$	$0.1648\pm 0.0083$	$0.1495\pm 0.0579$	$0.2312\pm 0.0135$	$0.1531\pm 0.0570$
5 ($P'$)	$\checkmark$	$\checkmark$	$\checkmark$	$0.1702\pm 0.0084$	$0.1612\pm 0.0522$	$0.2364\pm 0.0135$	$0.1650\pm 0.0514$
($P''$)	$\checkmark$	$\checkmark$	$\checkmark$	$0.1707\pm 0.0085$	$0.1648\pm 0.0539$	$0.2372\pm 0.0134$	$0.1685\pm 0.0532$

The formulas of errors are detailed in Sect. B (Eq. (S5)) of Supplementary Information. Remarks for Columns 1–4 and presented results of errors are the same as those in Table 4. In addition, the remarks for the choice of $\mu ^*=10^{1.8}$ in Model 5 and the partitions *P*, $P'$, and $P''$ are the same as those in Table 5.

The last three rows show the comparison of errors for Model 5 with three partitions *P*, $P'$, and $P''$ respectively. We may see that the testing errors of Model 5 with the incorrect partitions are basically the same and larger than those with the ground truth partitions. Nevertheless, these errors are still smaller than the other baseline models. Therefore, the incorrect division might worsen the generalization performance of Model 5; however, when the division does not deviate from the ground truth much, it still behaves better than Models 1–4 with uniform prior.

Moreover, as shown in Table 6, both Model 2, which allows transportation but not heterogeneity, and Model 3, that introduces heterogeneity but no transportation, behave worse than the baseline Model 1. Model 2 having more significant testing errors is due to the same reason as the case of four provinces, as has been analyzed in “Further model evaluation”. The reason for Model 3 not being able to predict well is explained in detail in “Results of trajectory prediction”.

However, we also note that Model 4, which involves both transportation and heterogeneity of parameters, has greater testing errors than Model 1 on average. This phenomenon may be partly explained by the fact that, on the one hand, there may be more infected cases imported from regions with higher $\lambda _k$’s to those with lower transmission rates. On the other hand, although the transmission parameters are the same and all-around 0.42 in Model 1, $E_0$ and $H_0$ are still allowed to be spatially heterogeneous, which makes the trajectories not that far away from the ground truth. Meanwhile, though the $\lambda _k$’s are allowed to be spatially heterogeneous in Model 4, it often underestimates $\lambda _k$’s for provinces in $D_1$ and overestimates $\lambda _k$’s for provinces in $D_3$, making the trajectories deviate from the ground truth.

Figure 7 and Supplementary Fig. S4 plot the change of weighted and simply averaged testing errors MAE$^{[\mathrm Te]}_{(w)}$, MSE$^{[\mathrm Te]}_{(w)}$, MAE$^{[\mathrm Te]}_{(s)}$ and MSE$^{[\mathrm Te]}_{(s)}$ regarding $\mu$ (recall that the subscripts (*w*) and (*s*) denote weighted and simple average respectively). We can still observe that Model 5 with the minimizer of validation errors also achieves the minimal testing errors, since the validation and testing errors have the same distribution for the simulated data.

## Experimental results on COVID-19 data

In this section, we apply our model to two real-world data sets, the COVID-19 data in China (from January to early February 2020) and Europe (from May to August 2020), in “Results for COVID-19 data in China” and “Results for COVID-19 data in Europe” respectively. We remark that the regions in “Methods” refer to provinces or municipalities for the data in China and refer to countries for the data in Europe. Before reporting results, we first introduce the data sources in “Data sources”. Then, we generalize our inference method to the case that the transmission rates have temporal heterogeneity in “Model extension by allowing time-varying parameters”, which might happen in real-world data due to travel restrictions or other reasons. For the two real-world data sets, we compare the proposed model with other baselines in the aspects of trajectory prediction and quantitative evaluation. We also show the estimation of transmission parameters with posterior distributions using our proposed model. Details can be found in “Results for COVID-19 data in China” and “Results for COVID-19 data in Europe”.

For the real-world data in Europe, since there is not consensus on proper divisions, we presented the results from two different partitions, both of which are based on geographical locations of the countries. The results imply that in comparison to the simulation data where the data are generated in the artificially divided regions, the generalization performances for the real-world data of both the partitions are similar and both much better than the baseline models.

### Data sources

Real-world data studied in this section involve the data in China and Europe.

#### Real-world data in China

The publicly available pandemic data in China include the number of confirmed cases and removed cases (consisting of recovered cases and fatalities) in China’s major provinces and municipalities from January 21st to March 28th, 2020. These publicly available data are downloaded from the websites of the National Health Commission of the People’s Republic of China^16^ and Chinese Center for Disease Control and Prevention^17^. The corresponding total population in each province or municipality is from Ref.^51^.

We also utilize two transportation data sets in China extracted from Baidu Qianxi^52^, lasting from January 10th to February 10th in 2020: (1) the migration indexes which reflects the number of people moving out from the provinces, and (2) the migration percentage which reflects the percentage of the population moving to a destination province from the origin province. The traveling volume is estimated by combining these two data sets.

#### Real-world data in Europe

The publicly available pandemic data in Europe include the number of confirmed and removed cases (consisting of recovered cases and fatalities) in the following 11 countries from May 1st to August 31st in 2020: Denmark, Finland, Norway, Austria, Germany, Switzerland, Italy, Spain, Belgium, France, and Ireland. The data are downloaded from Refs.^13,53^, where the data are collected from European Center for Disease Prevention and Control^18^. The data of total population in each country is obtained from Ref.^54^. During the study, we are not able to obtain suitable transportation data between these European countries needed by our model, and thus we simplify the proposed model by incorporating only spatial and temporal heterogeneity but no transportation in this experiment, see details in “Results for COVID-19 data in Europe”.

We remark that the data in China are from the initial outbreak of the epidemic. On the contrary, the data in Europe are from when the epidemic has been ongoing for about six months. The choice of the aforementioned time period of interest is to illustrate that the proposed model could predict both the case when the epidemic breaks out from some seed region and the case when the epidemic has already established a sizable local spread within different regions.

### Model extension by allowing time-varying parameters

To capture the trend that the transmission parameters might change in real-world data due to travel restrictions or other factors, we first extend the model and parameter estimation procedure described in “Methods” so that $\{\lambda _k\}$ are allowed to be time-varying.

Specifically, suppose that $\lambda _k$ is a piece-wise constant function of *t*, and $\lambda _k = \lambda _{k}^{(1)}$ for days before some threshold $T_k$, and $\lambda _k = \lambda _{k}^{(2)}$ afterwards.

We denote ${\lambda ^{(1)}} = (\lambda _{1}^{(1)},\dots ,\lambda _{n}^{(1)})^T$ and ${\lambda ^{(2)}} = (\lambda _{1}^{(2)},\dots ,\lambda _{n}^{(2)})^T$ as the vectors of transmission rates in the two periods respectively. The stochastic dynamic model introduced in “Model description” remains unchanged except that the transmission rates will vary as time increases.

As for the parameter estimation, the first step of obtaining $\gamma _k^* = \arg \max _{\gamma _k} \prod _{i=1}^T \text {Pois}\big ((\Delta R_a)_k(i) \big | ((C_a)_k(i-1) - (R_a)_k(i-1))\gamma _k\big )$ also stays the same as described in “Estimation of model parameters”.

Then, when estimating ${\overline{\Theta }} = \{E_k(0), H_k(0), \lambda _{k}^{(1)}, \lambda _{k}^{(2)}\}_{k=1}^n$ and the marginal posterior distributions of ${\overline{\Theta }}$, we modify the prior distribution $p({\overline{\Theta }})$ as below,

4.1 $$\begin{aligned} P({\overline{\Theta }}) = \frac{1}{C_{\sigma ,A}} \exp (-{\lambda ^{(1)}}^T (D-A){\lambda ^{(1)}} - \sigma \Vert {\lambda ^{(1)}}\Vert _2^2 - {\lambda ^{(2)}}^T (D-A){\lambda ^{(2)}} - \sigma \Vert {\lambda ^{(2)}}\Vert _2^2), \end{aligned}$$

where $\sigma$, *D* and *A* have the same meaning as in (2.10), and $C_{\sigma ,A} = \int _{\mathbb {R}^{2n}} \exp (-{\lambda ^{(1)}}^T (D-A){\lambda ^{(1)}} - \sigma \Vert {\lambda ^{(1)}}\Vert _2^2-{\lambda ^{(2)}}^T (D-A){\lambda ^{(2)}} - \sigma \Vert {\lambda ^{(2)}}\Vert _2^2) d{\lambda ^{(1)}}d{\lambda ^{(2)}}$ is still the normalizing constant. Consequently,

4.2 $$\begin{aligned} V({\overline{\Theta }})&=-\log \left( P\left( \left\{ (\Delta C_a)_k(i)\right\} _{k,i} \bigg | {\overline{\Theta }}\right) \right) - \log (P({\overline{\Theta }})) \nonumber \\&= -\log \left( P\left( \left\{ (\Delta C_a)_k(i)\right\} _{k,i} \bigg | {\overline{\Theta }}\right) \right) + {\lambda ^{(1)}}^T (D-A){\lambda ^{(1)}} +\sigma \Vert {\lambda ^{(1)}}\Vert _2^2 + {\lambda ^{(2)}}^T (D-A){\lambda ^{(2)}} \nonumber \\&\quad + \sigma \Vert {\lambda ^{(2)}}\Vert _2^2 + \log {C_{\sigma ,A}} \nonumber \\&= -\log \left( P\left( \left\{ (\Delta C_a)_k(i)\right\} _{k,i} \bigg | {\overline{\Theta }}\right) \right) + \frac{1}{2}\sum _{i,j} a_{ij} (\lambda _{i}^{(1)}-\lambda _{j}^{(1)})^2 + \sigma \Vert {\lambda ^{(1)}}\Vert _2^2 +\frac{1}{2}\sum _{i,j} a_{ij} (\lambda _{i}^{(2)}-\lambda _{j}^{(2)})^2\nonumber \\&\quad +\sigma \Vert {\lambda ^{(2)}}\Vert _2^2+ \log {C_{\sigma ,A}}. \end{aligned}$$

Therefore, by the definition of $a_{ij}$ in (2.12), the estimation of ${\overline{\Theta }}$ by minimizing $V({\overline{\Theta }})$ can be equivalently written as

4.3 $$\begin{aligned} {\overline{\Theta }}^*&= \arg \min V({\overline{\Theta }}) \nonumber \\&= \arg \min \bigg (-\log \left( P\left( \left\{ (\Delta C_a)_k(i)\right\} _{k,i} \bigg | {\overline{\Theta }}\right) \right) + \frac{1}{2}\sum _{i,j} a_{ij} (\lambda _{i}^{(1)}-\lambda _{j}^{(1)})^2 + \sigma \Vert {\lambda ^{(1)}}\Vert _2^2 \nonumber \\&\quad + \frac{1}{2}\sum _{i,j} a_{ij} (\lambda _{i}^{(2)}-\lambda _{j}^{(2)})^2 + \sigma \Vert {\lambda ^{(2)}}\Vert _2^2\bigg )\nonumber \\&{= \arg \min \Bigg (-\log \left( P\left( \left\{ (\Delta C_a)_k(i)\right\} _{k,i} \bigg | {\overline{\Theta }}\right) \right) + \frac{\mu }{2}\bigg (\sum _m\sum _{i,j\in D_m} W_{ij} \left( (\lambda _{i}^{(1)}-\lambda _{j}^{(1)})^2 + (\lambda _{i}^{(2)}-\lambda _{j}^{(2)})^2\right) }\nonumber \\&\quad {+ \beta \sum _{m_1<m_2}\sum _{i\in D_{m_1},j\in D_{m_2}}W_{ij} \left( (\lambda _{i}^{(1)}-\lambda _{j}^{(1)})^2 + (\lambda _{i}^{(2)}-\lambda _{j}^{(2)})^2\right) \bigg )+ \sigma \sum _i ((\lambda _{i}^{(1)})^2+(\lambda _{i}^{(2)})^2)\Bigg ).} \end{aligned}$$

After choosing *W* and $\sigma$, ${\overline{\Theta }}$ can be inferred through (4.3). Then, we could estimate the marginal posterior distributions of ${\overline{\Theta }}$ by MCMC sampling with the initial point ${\overline{\Theta }}^*$.

We would like to remark that same as in the simulation study, since the proposed model depends on the penalty factor $\mu$ as shown in (4.3), $\mu$ is chosen so that the corresponding validation error achieves or is slightly higher than the minimum. Specifically, results from the multiple choices of $\mu$ are present, since for the real-world data, the validation data may not have the same distribution as the testing data (the choice of the training, validation, and testing sets is detailed in Sects. A, D, and E of Supplementary Information.

### Results for COVID-19 data in China

#### Data description

After the selection process described in Sect. D of Supplementary Information, $n = 21$ provinces or municipalities are taken into consideration, which are Anhui, Beijing, Fujian, Gansu, Guangdong, Guangxi, Hebei, Henan, Hubei, Hunan, Jiangsu, Jiangxi, Liaoning, Ningxia, Shandong, Shan-Xi, Shanxi, Shanghai, Sichuan, Zhejiang, and Chongqing. Furthermore, since the epidemic data last from January 21st to March 28th, 2020, there are 20 days in total.

In addition, from observation of data from January 21st to February 10th, the transmission rates in the selected provinces or municipalities change after some specific time point. Thus, we allow $\{\lambda _k\}$ to be time-varying for this data set with appropriate changes to the model described in “Methods”, which are detailed in “Model extension by allowing time-varying parameters”. More details of the experimental settings can be found in Sect. D of Supplementary Information, including construction of training/validation/testing data and the choice of $T_k$, the day that $\lambda _k$ changes.

Furthermore, we remark that the data from Baidu Qianxi might not be the exact traveling volumes between municipalities and provinces. We assume that the actual traveling volume from one starting point to one destination is proportional to Baidu migration index (which reflects numbers of people departed from the stating point) and the percentage of population traveling from this origin to the destination. The corresponding scaling parameter $\alpha$ also needs to be inferred from the data for all the models.

#### Models to compare

As in “Models to compare”, the same Models 1–5 are compared for COVID-19 data in China. Recall that Model 5 is the model proposed in this paper and the other four are the baseline models for comparison.

For Model 5, the parameter inference adopts the method in “Estimation of model parameters”, while allowing $\{\lambda _k\}_{k=1}^n$ to be time-varying as described in “Model extension by allowing time-varying parameters”. Note that the medical resources were overwhelmed in Wuhan at the early stage of the pandemic^55^. When applying the general formula (4.3), we divide the provinces into two groups, which are Hubei and other provinces except Hubei. Furthermore, the affinity matrix *W* is constructed as averaged traveling volumes, that is $W_{ij} = {\bar{W}}_{ij} / \max _{i,j}{\bar{W}}_{ij}$, where ${\bar{W}}_{ij}=\frac{1}{2}(\frac{1}{T}\sum _{l=1}^T(w_l)_{ij} + (\frac{1}{T}\sum _{l=1}^T(w_l)_{ji})$. Then, for COVID-19 data in China, (4.3) can be specified as follows to estimate ${\overline{\Theta }}= \{E_k(0), H_k(0), \lambda _{k}^{(1)}, \lambda _{k}^{(2)}\}_{k=1}^n$ and scaling parameter $\alpha$:

4.4 $$\begin{aligned} {\overline{\Theta }}^*,\alpha ^*&= \arg \min \Bigg (-\log P\left( \left\{ (\Delta C_a)_k(i)\right\} _{k,i} \bigg | {\overline{\Theta }},\alpha \right) + \frac{\mu }{2} \bigg (\sum _{m=1}^2 W_{ij} \left( (\lambda _{i}^{(1)}-\lambda _{j}^{(1)})^2+(\lambda _{i}^{(2)}-\lambda _{j}^{(2)})^2\right) \nonumber \\&\quad + \beta \sum _{i\in D_1,j\in D_2} W_{ij} \left( (\lambda _{i}^{(1)}-\lambda _{j}^{(1)})^2 + (\lambda _{i}^{(2)}-\lambda _{j}^{(2)})^2\right) \bigg ) + \sigma \sum _i ((\lambda _{i}^{(1)})^2+(\lambda _{i}^{(2)})^2)\Bigg ), \end{aligned}$$

where we take $\beta =0.1, \sigma =10^{-6}$, and $\lambda _{k}^{(1)}$ and $\lambda _{k}^{(2)}$ are the transmission rates in the *k*-th province before and after the $T_k$-th day respectively.

For Models 1–4, if the model does not have heterogeneity of transmission parameters, then $\{\lambda _{1}^{(1)},\dots ,\lambda _{n}^{(1)}\}$ are forced to be the same and so do $\{\lambda _{1}^{(2)},\dots ,\lambda _{n}^{(2)}\}$, while the transmission rates in the two periods are allowed to be different; if the model does not allow transportation between provinces, then terms involving $W_t$ disappear in (2.4) as described in “Models to compare”.

#### Results of trajectory prediction

We first remark that for Model 5, three values of $\mu$ are chosen, and the trajectory prediction results presented below are from these three choices to perform the sensitivity analysis of $\mu$. The first value is $\mu ^*=10^{2.3}$, at which the relative validation errors ($\text {MAE}_{(w)}^{[\mathrm Val]}$, $\text {MSE}_{(w)}^{[\mathrm Val]}$, $\text {MAE}_{(s)}^{[\mathrm Val]}$, and $\text {MSE}_{(s)}^{[\mathrm Val]}$) are minimized, marked with blue squares in Fig. 12 and Supplementary Fig. S5. The other two values are $\mu =10^{1.7}$ and $\mu =10^{2.9}$ obtained by perturbing the minimizer $\mu ^*=10^{2.3}$, whose validation errors are slightly larger, marked with orange pentagrams and green diamonds in Fig. 12 and Supplementary Fig. S5 respectively. Since for the real-world data in China, the validation error does not necessarily have the same distribution as the testing error, the results from various choices of $\mu$ are presented for better comparison.

**Figure 8:**
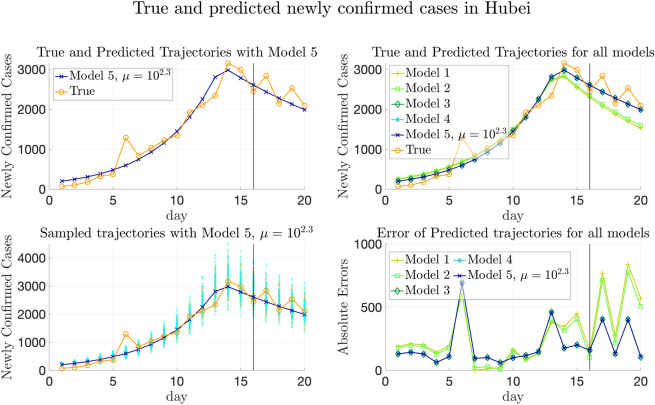
True and fitted trajectories in Hubei. The orange line with circles shows the true trajectory, the blue lines with crosses show the predicted deterministic trajectories using Model 5, blue and orange scatter plots show 100 stochastic trajectories with $\Theta ^*$ inferred using Model 5 and sampled from the posterior distribution of ${\overline{\Theta }}$ respectively. In each figure, the black vertical line shows the threshold of training-testing split. For Model 5, $\mu ^*=10^{2.3}$ is chosen, at which the validation errors achieves the minimum value, marked by blue squares in Fig. 12 and Supplementary Fig. S5.

Figure 8 shows the true and predicted trajectories for newly confirmed cases in Hubei. In Fig. 8,

- The orange line with circles shows the true trajectory.
- The blue lines with crosses show the predicted deterministic trajectories obtained by running (2.4) with $\Theta ^*$ inferred using (4.4) with $\mu ^*=10^{2.3}$. The predicted trajectories with $\mu =10^{1.7}$ and $10^{2.9}$ are close to the ones with $\mu ^*=10^{2.3}$, and are therefore not included in Fig. 8.
- The blue scatter plots show 100 stochastic trajectories with $\Theta ^*$ inferred using Model 5 with $\mu ^*= 10^{2.3}$.
- The orange scatter plots show 100 stochastic trajectories sampled from the posterior distribution of ${\overline{\Theta }}$, which is estimated by $5\times 10^5$ MCMC iterations staring from ${\overline{\Theta }}^*$ inferred using Model 5 with $\mu ^*=10^{2.3}$.

The deterministic trajectories obtained by the other four models and the corresponding absolute errors are also plotted for better comparison. It can be seen that all the predicted deterministic trajectories generally capture the trend of the true trajectory. Besides, it can be seen from Fig. 8 that the performances of all the models that include heterogeneity of parameters are similar for Hubei. This may be explained by the fact that the cases in Hubei outnumber those in other provinces or municipalities, which makes all the models tend to fit the trajectory of Hubei best. Moreover, sampling trajectories from the posterior distribution of ${\overline{\Theta }}$ generates more randomness than sampling trajectories with $\Theta ^*$.

**Figure 9:**
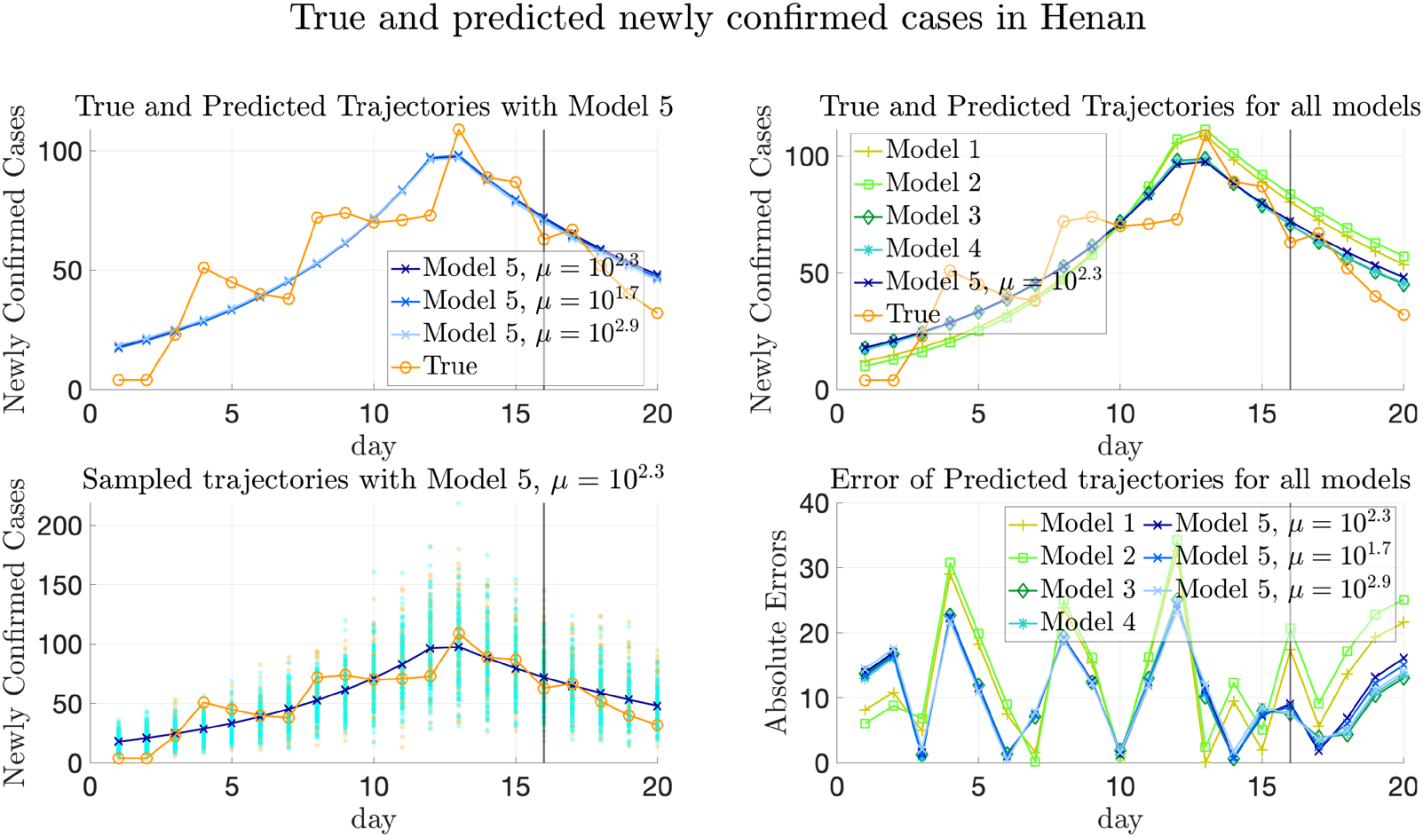
True and fitted trajectories in Henan. The remarks for the lines and scatter plots are the same as those in Figure 8. For Model 5, $\mu =10^{1.7}, 10^{2.3}$, and $10^{2.9}$ are chosen. As shown in Fig. 12 and Supplementary Fig. S5, the validation errors are minimized at $\mu ^*=10^{2.3}$. $\mu =10^{1.7}$ and $10^{2.9}$ are obtained by perturbing the minimizer $\mu =10^{2.3}$ without increasing validation errors much. $\mu =10^{1.7}, 10^{2.3}$, and $10^{2.9}$ are marked by yellow pentagrams, blue squares, and green diamonds respectively in Fig. 12 and Supplementary Fig. S5.

Similarly, Fig. 9 shows the predicted trajectories in Henan. When the transmission parameters are forced to be the same in all provinces, the increment of the newly confirmed cases of the predicted trajectory is faster than the trend shown in the true trajectory in the first period. Thus, heterogeneity helps improve the performance of fitting and predicting. Additionally, although the validation errors achieves the minimum at $\mu ^*=10^{2.3}$, it can be seen from Fig. 9 that the deterministic trajectory obtained by Model 5 with $\mu =10^{1.7}$ and $10^{2.9}$ achieves slightly better performance in prediction than the one obtained by Model 5 with $\mu ^*=10^{2.3}$. Therefore, the result suggests that the choice of $\mu$ is not necessarily limited to the minimizer of the validation error. Instead, we may also compare trajectories with $\mu$ that have slightly larger validation error for possibly better generalization performance.

**Figure 10:**
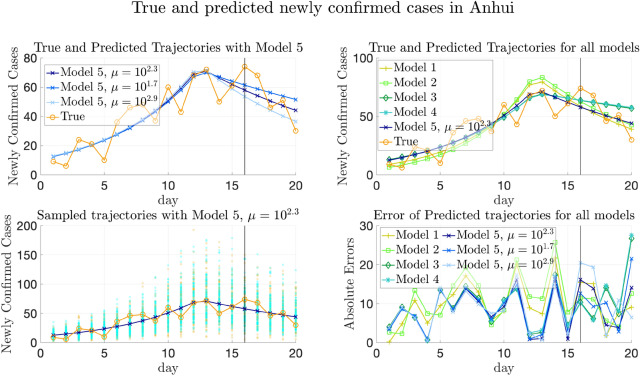
True and fitted trajectories in Anhui. The remarks for the lines and scatter plots are the same as those in Fig. 8. The choice of $\mu =10^{1.7},10^{2.3}$, and $10^{2.9}$ has been explained in Fig. 9.

Moreover, from Fig. 10 which shows the fitted trajectories in Anhui, it can be seen that heterogeneity helps the models fit the training trajectory better. Furthermore, adding regularization helps Model 5 capture the trend of the trajectory better than Model 4.

#### Results of parameter estimation

**Figure 11:**
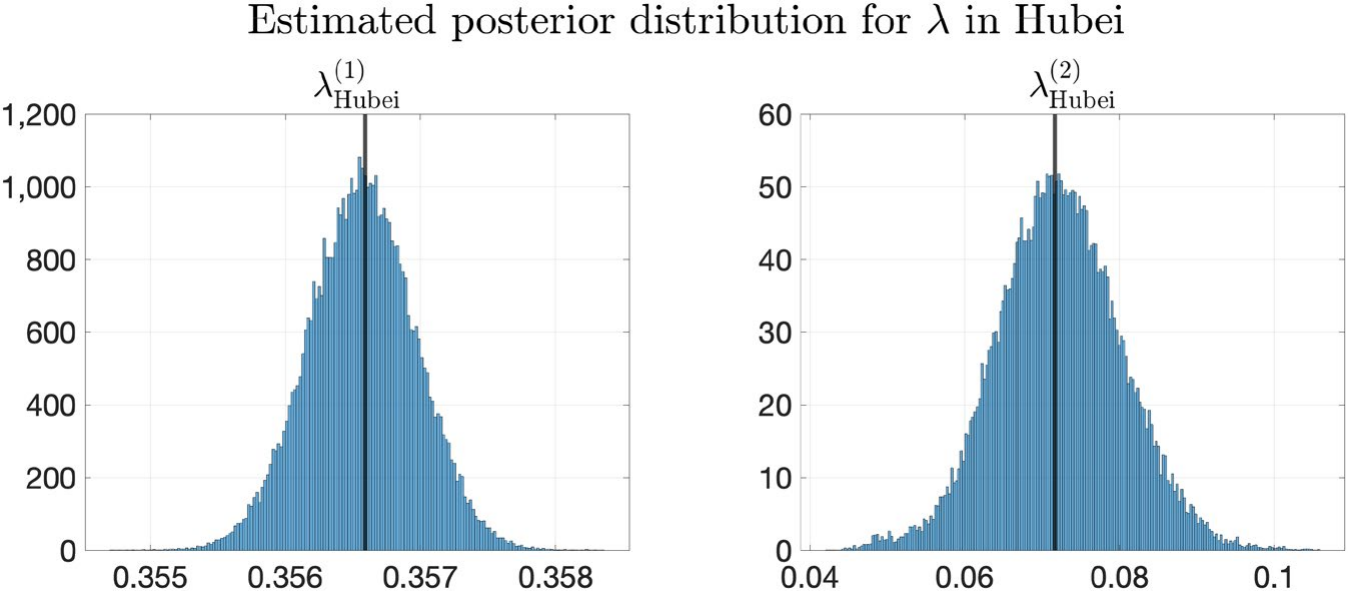
Estimated posterior distribution of $\lambda$ in Hubei. The vertical black lines represent the values of corresponding $\theta ^*$ using Model 5.

To illustrate the estimate of the posterior of parameters, Fig. 11 shows the estimated posterior distributions of $\lambda _{k}^{(1)}$ and $\lambda _{k}^{(2)}$ in Hubei using Model 5, in which the vertical black lines show the inferred $\lambda _{k}^{(1)}$ and $\lambda _{k}^{(2)}$ with $\mu =10^{2.3}$, and Table 7 shows the inferred $\lambda _{k}^{(1)}$ and $\lambda _{k}^{(2)}$ with the mean and standard deviation of their estimated posterior distributions from MCMC.

By comparing the transmission rates in Hubei in the two periods, it can be seen that the transmission rate decreases greatly after February 2nd, which indicates that the containment measures adopted in Hubei against the COVID-19 outbreak are effective.

**Table 7:** Estimated transmission rates in Hubei.

	$\theta ^*$	Mean of estimated posterior	Standard deviation of estimated posterior
$\lambda _\mathrm{Hubei}^{(1)}$	0.3566	0.3566	$3.9623\times 10^{-4}$
$\lambda _\mathrm{Hubei}^{(2)}$	0.0723	0.0716	0.0076

**Figure 12:**
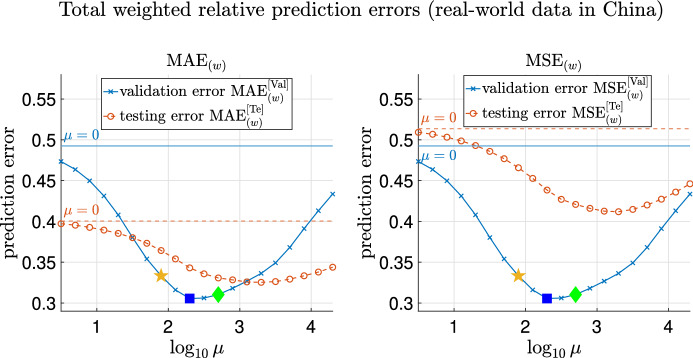
Testing and validation errors on real-world COVID-19 data in China. The two plots are similar to those in Figure 7, with errors computed on the real-world data in China instead of simulated data. Both the MAE and MSE validation errors are minimized at $\mu =10^{2.3}$, which is marked by blue squares in both plots. $\mu =10^{1.9}$ and $10^{2.7}$ are chosen by perturbing the minimizer $\mu =10^{2.3}$, have slightly larger validation errors, and are marked with yellow pentagrams and green diamonds in both plots. The construction of training/validation/testing data is detailed in Sects. A.1.2 and D of Supplementary Information.

$\theta ^*$ is inferred using (4.4) with $\mu =10^{2.3}$, at which the validation errors are minimized as shown in Fig. 12 and Supplementary Fig. S5. The posterior distribution is estimated by $5\times 10^5$ MCMC iterations.

#### Further model evaluation

Table 8 shows the training errors and testing errors of the five models computed as detailed in Sect. B of Supplementary Information. As can be seen from Table 8, both heterogeneity of parameters and transportation between provinces help reduce the training errors and the testing errors as the simulated data case. The utilization of graph Laplacian prior helps further reduce the testing errors. Note that the testing errors of Model 4 are close to those of Model 3, which might be explained as after the traveling restrictions are imposed on January 23rd^56^, transportation no longer has much influence on the spread of the epidemic.

**Table 8:** Training and testing errors of Models 1–5 for real-world data in China.

Model	M	H	GL prior	MAE$^{[\mathrm Tr]}_{(w)}$	MAE$^{[\mathrm Te]}_{(w)}$	MSE$^{[\mathrm Tr]}_{(w)}$	MSE$^{[\mathrm Te]}_{(w)}$
1	$\times$	$\times$	$\times$	0.399	0.497	0.650	0.626
2	$\checkmark$	$\times$	$\times$	0.388	0.459	0.645	0.590
3	$\times$	$\checkmark$	$\times$	0.296	0.398	0.565	0.511
4	$\checkmark$	$\checkmark$	$\times$	0.296	0.400	0.562	0.514
5	$\checkmark$	$\checkmark$	$\checkmark (\mu \,=10^{1.7})$	0.296	0.373	0.565	0.477
	$\checkmark$	$\checkmark$	$\checkmark (\mu ^*=10^{2.3})$	0.298	0.343	0.572	0.438
	$\checkmark$	$\checkmark$	$\checkmark (\mu \,=10^{2.9})$	0.303	0.328	0.582	0.416

The formulas of errors are detailed in Sect. B (Eq. (S5)) of Supplementary Information. The remarks for Columns 1–4 are the same as those in Table 4. In addition, the choice of $\mu =10^{1.7}, 10^{2.3}$, and $10^{2.9}$ in Model 5 has been explained in Fig. 9. Note that the validation errors are minimized at $\mu ^*=10^{2.3}$ as shown in Fig. 12.

Figure 12 and Supplementary Fig. S5 plot weighted and simply averaged testing errors against varying $\mu$ respectively. We can see from Table 8 and Fig. 12 that though $\mu ^*=10^{2.3}$ achieves the minimal validation error, it has larger testing error than $\mu =10^{2.9}$.

### Results for COVID-19 data in Europe

#### Data description

The dataset contains numbers of daily COVID-19 cases in $n=11$ countries in Europe, which are Denmark, Finland, Norway, Austria, Germany, Switzerland, Italy, Spain, Belgium, France, and Ireland. After preprosessing and removing the first and last three days of the original data, the dataset spans over a period of $T=116$ days. More details of experimental settings can be found in Sect. E of Supplementary Information, including the selection of the countries, the preprocessing of the data, the construction of training/validation/testing data, and the choice of the day that $\lambda _k$ changes in the selected countries.

#### Models to compare

**Three models for comparison.** For COVID-19 data in Europe, only three models detailed below are compared, which allow heterogeneity of parameters but do not include transportation. The last model is the proposed one in this paper, and the first two are baseline models. The transportation data needed in this model are not available to the best of our knowledge. However, the impact of transportation is expected to be less significant due to travel restrictions^56^.

1. Model with uniform prior distribution, without heterogeneity or migration.
2. Model with uniform prior distribution, with heterogeneity but without migration.
3. Model with prior distribution based on graph Laplacian, with heterogeneity but no migration.

**Two different partitions of the countries.** For Model 3’, 11 countries are partitioned into $d=4$ groups according to geographical locations:

- $D_1$ (Northern Europe): Denmark, Finland, Norway;
- $D_2$ (Central Europe): Austria, Germany, Switzerland;
- $D_3$ (Southern Europe): Italy, Spain;
- $D_4$ (Western Europe): Belgium, France, Ireland.

We denote this partition as *P*. Since this might not be the unique appropriate partition for the $n=11$ countries, we also present results from another partition denoted as $P'$ in the following sections. $P'$ also groups the countries that are geographically close together:

- $$D_1'$$: Finland, Norway;
- $D_2'$: Denmark, Austria, Germany;
- $D_3'$: Spain, Belgium, France, Ireland;
- $$D_4'$$: Switzerland, Italy.

The results for Model 3’ in “Further model evaluation” are presented with both the partitions *P* and $P'$.

**Parameter inference of the three models.** By the time-varying extension (4.3) detailed in “Model extension by allowing time-varying parameters”, for Model 3’, ${\overline{\Theta }}= \{E_k(0), H_k(0),\lambda _{k}^{(1)}, \lambda _{k}^{(2)}\}_{k=1}^n$ are estimated by the optimization problem in (4.5):

4.5 $$\begin{aligned} {\overline{\Theta }}^*&= \arg \min \Bigg (-\log P\left( \left\{ (\Delta C_a)_k(i)\right\} _{k,i} \bigg | {\overline{\Theta }}\right) + \frac{\mu }{2}\bigg (\sum _m\sum _{i,j\in D_m} \left( (\lambda _{i}^{(1)}-\lambda _{j}^{(1)})^2 + (\lambda _{i}^{(2)}-\lambda _{j}^{(2)})^2\right) \nonumber \\&\quad {+ \beta \sum _{m_1<m_2}\sum _{i\in D_{m_1},j\in D_{m_2}} \left( (\lambda _{i}^{(1)}-\lambda _{j}^{(1)})^2 + (\lambda _{i}^{(2)}-\lambda _{j}^{(2)})^2\right) \bigg )+ \sigma \sum _i \left( (\lambda _{i}^{(1)})^2+(\lambda _{i}^{(2)})^2\right) \Bigg )}, \end{aligned}$$

or by (4.5) but with the partition $P=\{D_1,D_2,D_3,D_4\}$ replaced by the partition $P'=\{D_1',D_2',D_3',D_4'\}$. Here, we still take $\sigma =10^{-6}, \beta =0.1$.

#### Results of trajectory prediction

We still remark that for Model 3’ (with *P*), as in the case of real-world data in China, part of the results reported are from three choices of $\mu$ for careful consideration, since the validation error may not have the same distribution as the testing error. Note that Fig. 17 and Supplementary Fig. S6 plot the weighted and simply averaged relative validation errors, $\text {MAE}_{(w)}^{[\mathrm Val]}$, $\text {MSE}_{(w)}^{[\mathrm Val]}$, $\text {MAE}_{(s)}^{[\mathrm Val]}$ and $\text {MSE}_{(s)}^{[\mathrm Val]}$ respectively. One choice is $\mu ^*=10^{5.5}$, at which the validation errors (computed with *P*) achieve the minimum, marked with blue squares in Fig. 17 and Supplementary Fig. S6. The other two choices are $\mu =10^{5.2}$ and $\mu =10^{5.8}$, which have larger validation errors and are marked in orange pentagrams and green diamonds respectively in Fig. 17 and Supplementary Fig. S6.

**Figure 13:**
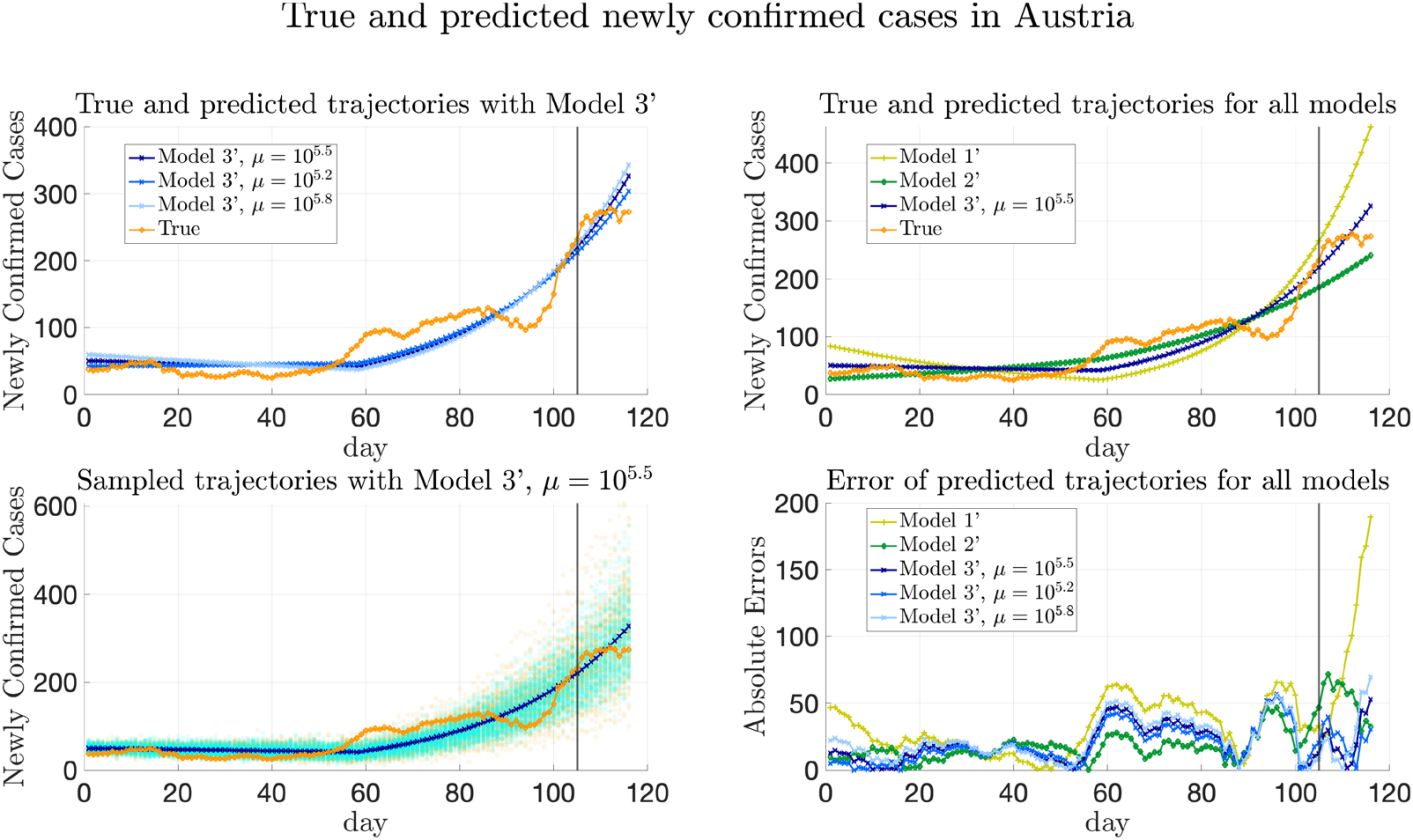
True and fitted trajectories in Austria. The remarks for the lines and scatter plots are the same as those in Fig. 8. The vertical lines show the threshold $T_{th}=102$ of training-testing split of COVID-19 data in Europe. For Model 3’, $\mu =10^{5.2}, 10^{5.5}$, and $10^{5.8}$ are chosen. As shown in Fig. 17 and Supplementary Fig. S6, the validation errors are minimized at $\mu =10^{5.5}$, and $\mu =10^{5.2}$ and $10^{5.8}$ are obtained by perturbing the minimizer $\mu =10^{5.5}$. $\mu =10^{5.2}, 10^{5.5}$, and $10^{5.8}$ are marked by yellow pentagrams, blue squares, and green diamonds respectively in Fig. 17 and Supplementary Fig. S6.

**Figure 14:**
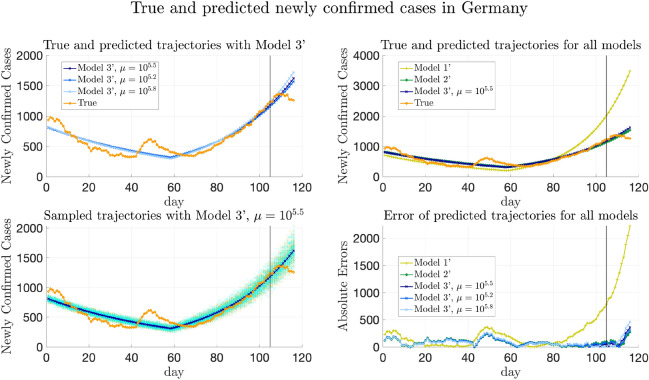
True and fitted trajectories in Germany. The remarks for the lines and scatter plots are the same as those in Fig. 8. The choice of $\mu =10^{5.2}, 10^{5.5}$, and $10^{5.8}$ has been explained in Fig. 13.

**Figure 15:**
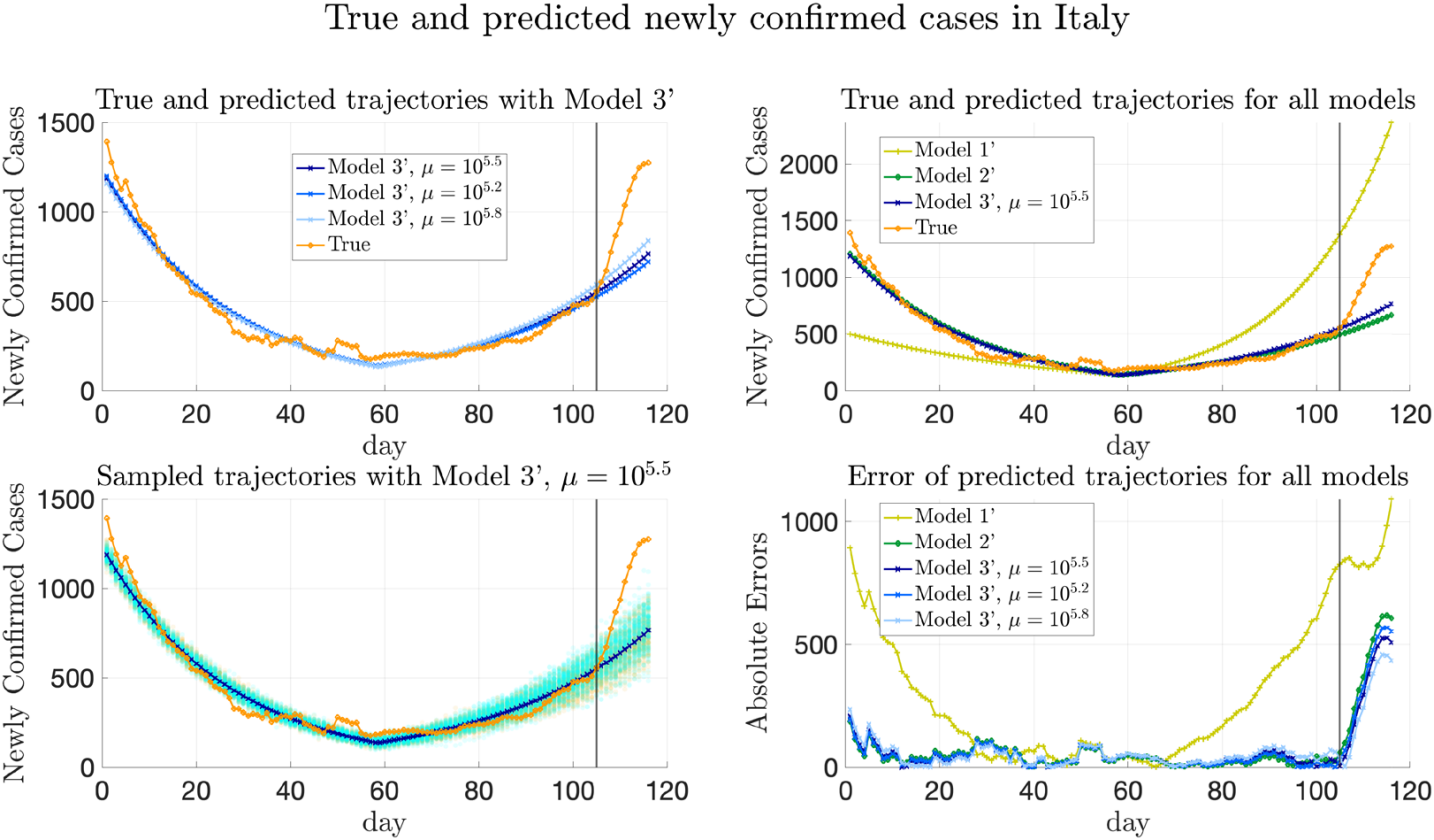
True and fitted trajectories in Italy. The remarks for the lines and scatter plots are the same as those in Fig. 8. The choice of $\mu =10^{5.2}, 10^{5.5}$, and $10^{5.8}$ has been explained in Fig. 13.

Figures 13, 14 and 15 present the true and predicted trajectories in Austria, Germany, and Italy, respectively. As can be seen from Figs. 13, 14 and 15, heterogeneity of transmission parameters in Model 2’ (green lines) and Model 3’ (blue lines) helps improve the performance of fitting and generalization. Furthermore, utilization of correlation between countries in Model 3’ further improves the prediction of the trajectories as shown in Fig. 13. We also note that for Model 3’, compared to the results for $\mu ^*=10^{5.5}$, at which the validation errors are minimized, $\mu =10^{5.2}$ and $10^{5.8}$ achieve slightly more accurate prediction for Italy.

#### Results of parameter estimation

**Figure 16:**
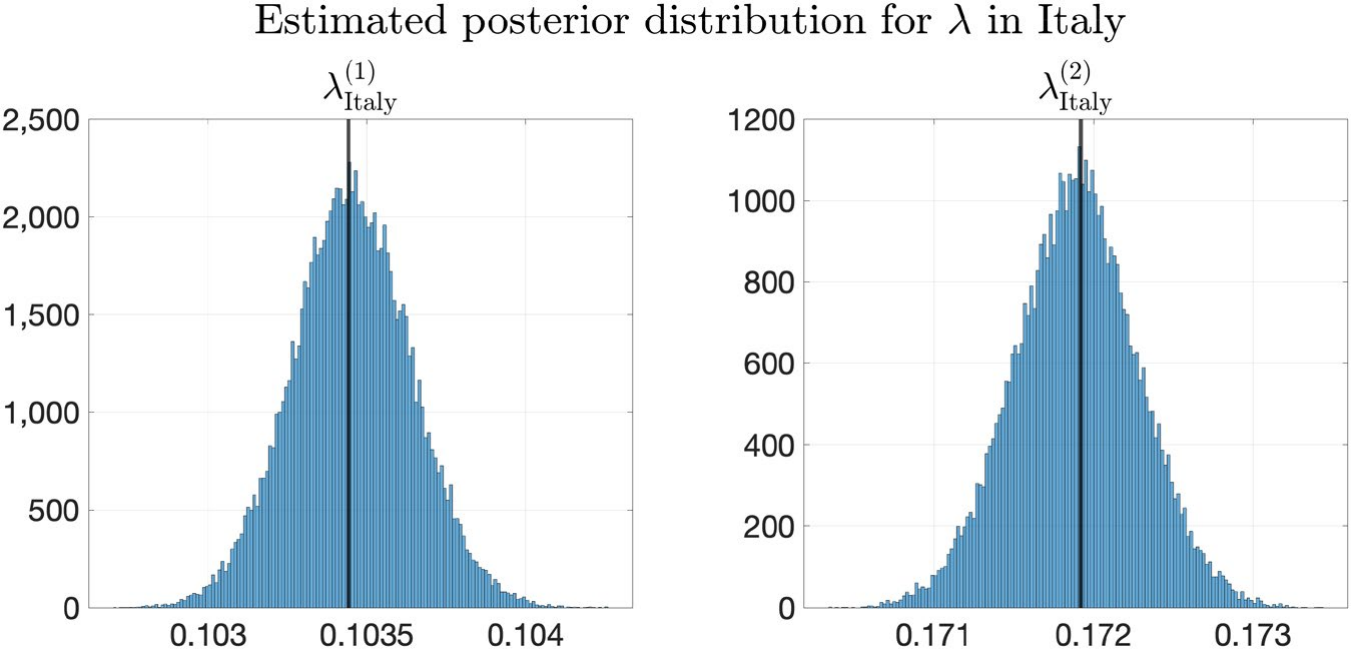
Estimated posterior distributions of $\lambda$ in Italy. The vertical black lines represent the values of corresponding $\theta ^*$ using Model 3’.

Same as before, Fig. 16 shows the estimated posterior distributions of $\lambda _\mathrm{Italy}^{(1)}$ and $\lambda _\mathrm{Italy}^{(2)}$ using Model 3’, in which the vertical black lines show the inferred $\lambda _\mathrm{Italy}^{(1)}$ and $\lambda _\mathrm{Italy}^{(2)}$ with $\mu ^*=10^{5.5}$. Table 9 shows the inferred $\lambda _\mathrm{Italy}^{(1)}$ and $\lambda _\mathrm{Italy}^{(2)}$ and the mean and standard deviation of their estimated posterior distribution from MCMC. The estimated $\lambda _\mathrm{Italy}^{(2)}$ is larger than the estimated $\lambda _\mathrm{Italy}^{(1)}$, which is consistent with the trend that the newly confirmed cases in Italy first decrease and then increase.

**Table 9:** Estimated transmission rates in Italy.

	$\theta ^*$	Mean of estimated posterior	Standard deviation of estimated posterior
$\lambda _\mathrm{Italy}^{(1)}$	0.1034	0.1035	$1.8768\times 10^{-4}$
$\lambda _\mathrm{Italy}^{(2)}$	0.1719	0.1719	$3.8791\times 10^{-4}$

**Figure 17:**
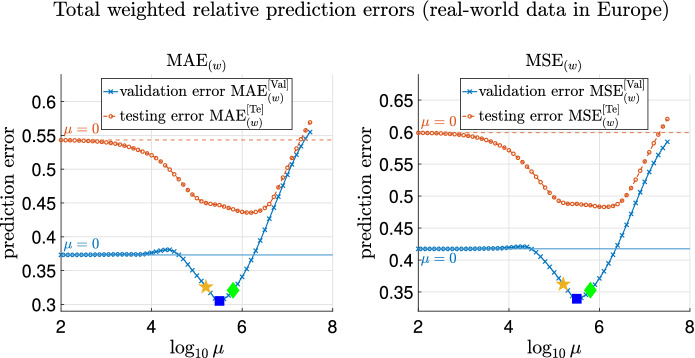
Testing and validation errors on real-world COVID-19 data in Europe. The two plots are similar to those in Fig. 7, with the errors computed on the real-world data in Europe instead of simulated data. Both the MAE and MSE validation errors are minimized at $\mu =10^{5.5}$, which is marked by blue squares in both plots. $\mu =10^{5.2}$ and $10^{5.8}$ are chosen by perturbing the minimizer $\mu =10^{5.5}$, have slightly larger validation errors, and are marked with yellow pentagrams and green diamonds in both plots. The construction of training/validation/testing data is detailed in Sects. A.1.2 and E of Supplementary Information.

$\theta ^*$ is inferred using (4.5) with $\mu =10^{5.5}$, at which the validation errors are minimized as shown in Fig. 17 and Supplementary Fig. S6. The posterior distribution is estimated by $5\times 10^5$ MCMC iterations.

#### Further model evaluation

Table 10 shows training and testing errors of the three models. It can be seen from Table 10 that Models 2’ and 3’ have smaller both training and testing errors than Model 1’, due to the introduction of heterogeneity of transmission parameters. We can also see that compared with Model 2’, Model 3’ that adds Graph Laplacian prior has better generalization performance. Especially, Model 3’ with $\mu =10^{5.8}$ has smaller testing error than with $\mu =10^{5.5}$, although $\mu =10^{5.8}$ does not achieve the minimal validation errors.

Recall that *P* and $P'$ are two different partitions of countries in Europe introduced in “Models to compare”, both based on geographical locations. By comparing the corresponding results in Table 10 for Model 3’ with partitions *P* and $P'$ respectively, it can be seen that the testing errors are close. This is different from the implications of the simulated data. A possible explanation for this might be that the regions in the real world have more complicated correlations than the simulated regions where clustering information is uniquely and artificially prefixed. Although reasonable groupings may not always be unique for real-world cases, the proposed model could still predict the trajectories more accurately than the baseline models.

**Table 10:** Training and testing errors of Models 1’–3’ for real-world data in Europe.

Model	M	H	GL prior	MAE$^{[\mathrm Tr]}_{(w)}$	MAE$^{[\mathrm Te]}_{(w)}$	MSE$^{[\mathrm Tr]}_{(w)}$	MSE$^{[\mathrm Te]}_{(w)}$
1’	$\times$	$\times$	$\times$	0.330	0.639	0.424	0.691
2’	$\times$	$\checkmark$	$\times$	0.171	0.543	0.228	0.599
3’ (*P*)	$\times$	$\checkmark$	$\checkmark (\mu \,=10^{5.2})$	0.203	0.450	0.268	0.489
	$\times$	$\checkmark$	$\checkmark (\mu ^*=10^{5.5})$	0.214	0.447	0.285	0.488
	$\times$	$\checkmark$	$\checkmark ((\mu \,=10^{5.8})$	0.225	0.441	0.302	0.486
3’ ($P'$)	$\times$	$\checkmark$	$\checkmark (\mu \,=10^{5.2})$	0.209	0.436	0.273	0.511
	$\times$	$\checkmark$	$\checkmark (\mu ^*=10^{5.5})$	0.222	0.415	0.291	0.492
	$\times$	$\checkmark$	$\checkmark (\mu \,=10^{5.8})$	0.237	0.399	0.311	0.480

The formulas of errors are detailed in Sect. B (Eq. (S5)) of Supplementary Information. The remarks for Columns 2–4 are the same as those in Table 4. In addition, the choice of $\mu =10^{5.2}, 10^{5.5}$, and $10^{5.8}$ in Model 3’ has been explained in Fig. 13. Note that the validation errors are minimized at $\mu ^*=10^{5.5}$ as shown in Fig. 17. The partitions in Model 3’ are *P* and $P'$ respectively. *P* and $P'$ are two different partitions of countries in Europe introduced in “Models to compare”.

Figure 17 below and Supplementary Fig. S6 plot the weighted and simply averaged testing errors (whose definitions are in Supplementary Information) against varying $\mu$ respectively, from which one may see that by imposing regularization properly through choosing a moderate $\mu$ helps improve the generalization performance. By comparing the blue solid lines and red dashed lines in Fig. 17 (and Supplementary Fig. S6), we see that the change in validation and testing errors concerning increasing $\mu$ are not the same. This further verifies the indications from the previous findings that it would be better to examine and compare the results from multiple choices of $\mu$ with relatively low validation errors for Model 3’ to choose a better $\mu$ for the specific data sets.

## Discussion

In this paper, we propose a stochastic dynamic model inspired by Ref.^2^, with considerations on the inter-district transportation and as well as spatially and temporally heterogeneous transmission parameters, which can model the ongoing and lasting spread of the epidemic in multiple districts. Based on the proposed model, we also introduce a two-step procedure for estimating the parameters, which utilizes graph Laplacian regularization to address the correlation between districts. Experiments on both simulated and real-world data show that compared with the baseline models, this proposed model improves the performance of fitting and generalization.

We acknowledge that there are limitations to this work. First, the stochastic dynamic model proposed in this paper might not be comprehensive enough to characterize the spread of COVID-19 in reality. For example, the model does not account for the ascertainment rate of the positive cases, the asymptomatic virus carriers, and the infectious latent period. Second, the proposed model does not consider the heterogeneity with respect to transmission risk in different populations, for example, populations with different ages, jobs, or health conditions, and needs further modification to incorporate the large-scale COVID-19 vaccination as well as waning of immunity over time. Finally, the parameter inference depends on the correlation between regions determined by the clustering patterns among regions. However, such division is usually not fully known in the real-world cases, and the inference of graph structure is yet to be explored in this paper.

The current methods have possible extensions in the following several directions in our future study. First, the dynamic model could be refined to be closer to reality, for example, taking the asymptomatic virus carriers and contact tracking into account and modeling the change of the transmission parameters over time in a more sophisticated way. Second, the model can be modified to accommodate the heterogeneity of various populations, including different age groups and vaccination statuses. For example, the compartments are to be further divided into subgroups, and the parameters such as transmission rates and mortality rates are allowed to be different; and the dynamic model is also to be adjusted accordingly. Third, the method could further include detecting the underlying graph structure of the regions so that the construction of the graph Laplacian matrix could be more self-contained and systematic. At last, the method could be further used to assess the influence of containment measures taken by different countries, for example, by adjusting the traveling volume to make it different from the actual transportation data and then analyzing the corresponding influence on the size and trend of the pandemic.

## Supplementary Information

Supplementary Information.

## Data Availability

The data and code used in the simulation study (“Experimental results for simulated data”) and the application to COVID-19 data in Europe (“Results for COVID-19 data in Europe”) are publicly available in the repository https://github.com/Yixuan-Tan/Statistical_Inference_Using_GLEaM_with_Spatial_Heterogeneity_and_Correlation. The COVID-19 data in China (“Results for COVID-19 data in China”) are not publicly available due to that the official websites of health commissions of some provinces in China no longer maintain the COVID-19 data before March of 2020, but are available from the corresponding author on request.
