# Supplementary material for: Statistical inference using GLEaM model with spatial heterogeneity and correlation between regions
Source: Sci Rep. 2022 Oct 5;12:16630. doi: 10.1038/s41598-022-18775-8 (PMC9534028; doi:10.1038/s41598-022-18775-8)
Supplement: Supplementary file 1 — Supplementary Information. [file 41598_2022_18775_MOESM1_ESM.pdf]

Statistical Inference Using GLEaM Model with Spatial Heterogeneity and Correlation between Regions

Yixuan Tan, Yuan Zhang, Xiuyuan Cheng, Xiao-Hua Zhou

Supplementary Information

A Data Splitting and Choice of Hyper-parameters

In this section, we first specify the choice of training, validation, and testing data, and explain the choice of the hyper-parameter μ through cross-validation in more detail for simulated and real-world data in Section A.1. Then, we detail the choice of the algorithmic hyper-parameters in Section A.2, including σ in (2.10), the penalty factor μ, the parameter $\beta \in (0, 1)$ in (2.12), and partition of regions.

A.1 Construction of Training/Testing/Validation Data

In this section, we detail how the training, testing, and validation data are chosen for the simulated and real-world study in Sections A.1.1 and A.1.2 respectively. Then, we also explain how the fitted/predicted trajectories are computed for the training/testing/validation data. The corresponding errors are calculated as the (weighted) average of daily relative errors for the fitted/predicted trajectories compared with the ground truth data.

We remark that the choice of training and testing data are for evaluating the model performance through training and testing errors. In addition, choosing validation data aims to help select hyper-parameters in the proposed model through validation errors, as will be mentioned in Section A.2. The details of computation of errors can be found in Section B.

A.1.1 Simulated Data

For simulated data, three random trajectories are sampled independently according to the stochastic model with prefixed parameters in one replica. For some threshold T_{th}, data from the first T_{th} days of one trajectory are treated as the training data $Y^{[\mathrm{Tr}]} = \{(\Delta C_a)_k^{[\mathrm{Tr}]}(i), k = 1, \dots, n\}_{i=1}^{T_{th}}$, data from the last $T - T_{th}$ days of the second trajectory are treated as the validation data $Y^{[\mathrm{Val}]} = \{(\Delta C_a)_k^{[\mathrm{Val}]}(i), k = 1, \dots, n\}_{i=T_{th}+1}^{T}$, and data from the last $T - T_{th}$ days of the last trajectory are treated as the testing data $Y^{[\mathrm{Te}]} = \{(\Delta C_a)_k^{[\mathrm{Te}]}(i), k = 1, \dots, n\}_{i=T_{th}+1}^{T}$. This guarantees that the training and validation data are independent from the testing data and that validation data have the same distribution as testing data.

The fitting performance is assessed by comparing the training data $Y^{[\mathrm{Tr}]}$ with the fitted deterministic trajectory $Y^{[\mathrm{Tr},\Theta]} = \{(\widetilde{\Delta C_a})_k^{[\mathrm{Tr},\Theta]}(i), k = 1, \dots, n\}_{i=1}^{T_{th}}$. $Y^{[\mathrm{Tr},\Theta]}$ is obtained by integrating ODE system (2.4) for T_{th} days with parameters $\Theta = \{E_k(0), H_k(0), \lambda_k, \gamma_k\}_{k=1}^{n}$ inferred by the corresponding model. The model can be either any baseline model or the newly proposed model.

Similarly, the generalization performance is assessed by comparing the testing data $Y^{[\mathrm{Te}]}$ with the predicted deterministic trajectory $Y^{[\mathrm{Te},\Theta]} = \{(\widetilde{\Delta C_a})_k^{[\mathrm{Te},\Theta]}(i), k = 1, \dots, n\}_{i=T_{th}+1}^{T}$. $Y^{[\mathrm{Te},\Theta]}$ is computed by running (2.4) for T days with parameters $\Theta = \{E_k(0), H_k(0), \lambda_k, \gamma_k\}_{k=1}^{n}$ and then taking out the data of the last $T - T_{th}$ days.

Moreover, to help determine the hyper-parameter in the proposed model, we evaluate the model's performance with parameters fitted using training data on the validation data set. Specifically, for a given hyper-parameter, we first fit the parameters $\Theta = \{E_k(0), H_k(0), \lambda_k, \gamma_k\}_{k=1}^{n}$ by the training data $Y^{[\mathrm{Tr}]}$ and (2.13). Then, we compute the predicted deterministic trajectory for the validation data, $Y^{[\mathrm{Val},\Theta]} =$

$\{(\Delta C_a)_k^{[\mathrm{Val},\Theta]}(i), k = 1, \ldots, n\}_{i=T_{th}+1}^{T}$, by integrating ODE system (2.4) for T days with the fitted Θ_μ and taking out the data of the last $T - T_{th}$ days. An illustrating plot is shown in Supplementary Figure S1b.

A.1.2 Real-world Data

For real-world data, only one observable trajectory is available, thus training, validation, and testing data ($Y^{[\mathrm{Tr}]}$, $Y^{[\mathrm{Val}]}$ and $Y^{[\mathrm{Te}]}$) are the three parts of the true trajectory split by two threshold, the $T_{th,1}$-th day and T_{th}-th day. Namely, we treat the data of the first $T_{th,1}$ days as training data $Y^{[\mathrm{Tr}]}$, the data of the $(T_{th,1}+1)$-th day to the T_{th}-th day as the validation data $Y^{[\mathrm{Val}]}$, and the data of the last $(T - T_{th})$ days as testing data $Y^{[\mathrm{Te}]}$. For the choice of hyper-parameters, the model parameters are first fit using the data from the first $T_{th,1}$ days, then the validation error is computed by comparing the validation data $Y^{[\mathrm{Val}]}$ with the predicted trajectory using the fitted parameters. For trajectory prediction and quantitative model evaluation, the model parameters are fit using the data from all the first T_{th} days (with the hyper-parameter chosen according to the validation error), and then the testing data $Y^{[\mathrm{Te}]}$ are predicted.

An illustrating plot is shown in Supplementary Figure S1a. We remark that for real-world data, $T_{th,1}$ should not be earlier than the maximum of T_k, which is the day that λ_k changes in the k-th region, $\forall k = 1, \ldots, n$.

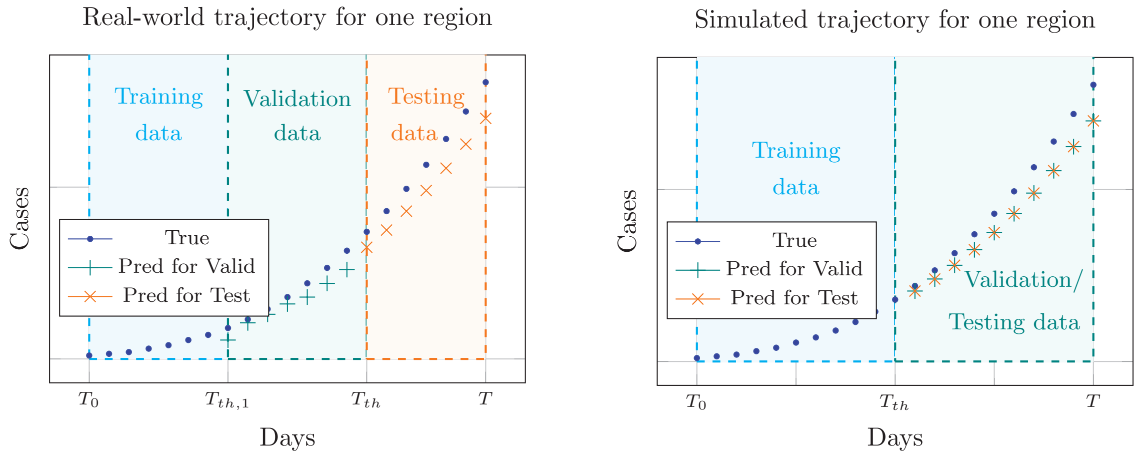

(a) Real-world data

(b) Simulated data

Supplementary Figure S1: Sketch maps illustrating how the training/validation/testing data are constructed for real-world/simulated data as described in Section A.1 of Supplementary Information. (a) For real-world case, only one trajectory is available, which is split into three parts: training/validation/testing data (see details in Section A.1.2 of Supplementary Information). (b) For simulated study, three trajectories are i.i.d. randomly generated, part of which are treated as training, validation, or testing data separately (see details in Section A.1.1 of Supplementary Information).

A.2 Choice of Hyper-parameters

It can be seen from Section 2.3 and Algorithm 1 that there are several hyper-parameters that determine the model proposed, including σ in the prior distribution (2.10) along with the factor μ for graph Laplacian

penalty, β, and partition of regions that all decide the affinity A through (2.12). We remark on the choice of these hyper-parameters as follows.

- σ: the factor for regularization on l_2 norm of the vector(s) of transmission parameters, appearing in both (2.13) and (4.3). In this paper, σ is chosen to be 10^{-6} for all the data sets. We present the results of sensitivity analysis performed for σ on the simulated data with four provinces in Section 3.2. The results are not sensitive to the choice of σ so long as σ is not too large. Similar patterns show for other data sets, thus details are not reported repetitively and the choice of σ is fixed as 10^{-6} throughout the experiments.

- Partition of regions: the partition determining construction of the affinity matrix A, such that correlation between regions from the same group in the partition is more addressed. For simulated data, experimental results are mainly reported with underlying graph information known. For real-world data, the partition of provinces in China is based on the medical overrun in Hubei at the initial outbreak of the epidemics, and the partition of countries in Europe is grounded on the geographical locations. Moreover, to test whether the model works when the graph information is not fully known, we also present results from mismatched/alternative partitions for simulated/real-world data in Section 3.3 and 4.4. The results show that the proposed model still predicts more accurately than the baseline models if the mismatch is not too serious (for the simulated data) or the reasonable partition may not be unique (for the real-world data).

- μ: the penalty factor of the graph Laplacian regularization, appearing in both (2.13) and (4.3). μ is chosen through cross-validation. Specifically, we separate out some validation data, then fit parameters Θ_μ for each μ and compute validation errors by comparing the actual validation trajectories with the predicted ones with fitted Θ_μ. Then, the choice of μ depends on the validation errors. In the Sections A.1.1 and A.1.2 above, we have specified how to choose validation set for simulated and real-world data in detail respectively. In addition, the formulas of validation errors can be found in Section B. For the case of simulated data, validation errors are identically distributed as testing errors, which are used to evaluate the prediction performance of models, μ is chosen to minimize the validation errors. On the contrary, for real-world data when validation errors may have different distribution from testing errors, results from multiple choices of μ with relative small validation errors are presented. Details of results are in corresponding sections of experimental results.

- β: the parameter taking values in $(0, 1)$ that reduces the correlation between inter-group regions through (2.12). In this paper, β is fixed to be 0.1 for all the experiments. We remark that other reasonable choices of $\beta \in (0, 1)$ would also lead to similar results, which might not be exactly the same as those with $\beta = 0.1$ though. β can also be chosen through cross-validation, similarly to the choice of μ, and we omit the details in this paper.

B Model Evaluation Metric

In Section A, we have specified the choice of ground truth training/testing/validation data and explained how they are estimated using models for both simulated and real-world data.

In the current subsection, we define the training, validation, and testing errors for model evaluation and help choose hyper-parameters of the model. Specifically, we introduce the definitions of mean relative error (which will be abbreviated as MAE hereinafter) and root mean squared relative error (which will be abbreviated as MSE for short), simply averaged or weighted.

In the paper, the superscripts $^{[\text{Tr}]}$, $^{[\text{Val}]}$, and $^{[\text{Te}]}$ refer to when the error (MAE and MSE) is computed on the training, validation and testing data respectively. We thus introduce the definition of error here omitting the superscript for simplicity.

We compute the error over the days from $i = 1$ to T_e. Given the data of true daily confirmed cases ΔC_a and those estimated from the model $\widetilde{\Delta C_a}$, we define

$$\text{Err}_k(i) = \frac{|(\Delta C_a)_k(i) - (\widetilde{\Delta C_a})_k(i)|}{(\Delta C_a)_k(i)} \tag{S1}$$

as the relative error in the k-th region on the i-th day. Then, the total error for the k-th region are the weighted sum of $\{\text{Err}_k(i)\}_{i=1}^{T_e}$. We use weighted average in computing the error, and adopt the following two types of weights $\alpha_k(i)$ cross region k and time i. The first type of weight, denoted with subscript $_{(w)}$, is defined as

$$\alpha_{(w),k}(i) = \frac{(\Delta C_a)_k(i)}{\sum_{i'=1}^{T_e} (\Delta C_a)_k(i')}, \tag{S2}$$

which share the same trend of increasing or decreasing with the newly confirmed cases in the k-th region and give larger weights to errors with more cases. The second type of weight conducts simple average over time, namely,

$$\alpha_{(s),k}(i) = \frac{1}{T_e}, \quad \forall k \in \{1, \ldots, n\}, \quad i \in \{1, \ldots, T_e\}. \tag{S3}$$

With the two types of weights (S2) and (S3), we define the weighted MAE and MSE for the k-th region as

$$\text{MAE}_{(w),k} = \sum_{i=1}^{T_e} \alpha_{(w),k}(i)\text{Err}_k(i), \quad \text{MSE}_{(w),k} = \left(\sum_{i=1}^{T_e} \alpha_{(w),k}(i) \left(\text{Err}_k(i)\right)^2 \right)^{1/2}. \tag{S4}$$

The errors weighted by $\alpha_{(s),k}(i)$ are defined similarly and denoted with subscript $_{(s)}$ instead. Note that, strictly speaking, the error for each day and region before taking average is a relative error, c.f. (S1). We use the name 'MAE' and 'MSE' following the convention.

The definition (S4) is for each region k. The total error over all regions is defined by taking an average over regions, that is,

$$\text{MAE}_{(w)} = \frac{1}{n} \sum_{k=1}^{n} \text{MAE}_{(w),k}, \quad \text{MSE}_{(w)} = \frac{1}{n} \sum_{k=1}^{n} \text{MSE}_{(w),k}. \tag{S5}$$

The simple-average weight counterparts $\text{MAE}_{(s)}$ and $\text{MSE}_{(s)}$ are defined in the same way from $\text{MAE}_{(s),k}$ and $\text{MSE}_{(s),k}$ respectively.

C Group Assignment and Transmission Parameters of Simulated Data with Thirty Provinces

For each of the thirty provinces, we randomly assign it into one of the $d = 3$ subgroups with equal probability. As a result, Group 1 (denoted as D_1) includes 9 provinces, indexed by Provinces 1-9; Group 2 (denoted as D_2) includes 12 provinces, indexed by Provinces 10-21; Group 3 (denoted as D_3) includes 9 provinces, indexed by Provinces 22-30. We denote this ground truth partition as P. Moreover, the transmission rates of provinces in the 3 groups are randomly sampled from normal distribution with mean 0.3, 0.4, and 0.5 respectively and all with standard deviation 0.01.

The choice and values of the transmission rates in the thirty provinces are listed below.

- For provinces in Group 1 (λ_k's are independently randomly sampled from Normal distribution with mean 0.3 and standard deviation 0.01):

  $\lambda_1 = 0.307, \lambda_2 = 0.295, \lambda_3 = 0.303, \lambda_4 = 0.299, \lambda_5 = 0.296,\ \lambda_6 = 0.296, \lambda_7 = 0.294, \lambda_8 = 0.293, \lambda_9 = 0.305$.

- For provinces in Group 2 (λ_k's are independently randomly sampled from Normal distribution with mean 0.4 and standard deviation 0.01):

  $\lambda_{10} = 0.416, \lambda_{11} = 0.410, \lambda_{12} = 0.405, \lambda_{13} = 0.393, \lambda_{14} = 0.400, \lambda_{15} = 0.402, \lambda_{16} = 0.406, \lambda_{17} = 0.407, \lambda_{18} = 0.395, \lambda_{19} = 0.410, \lambda_{20} = 0.396, \lambda_{21} = 0.419$.

- For provinces in Group 3 (λ_k's are independently randomly sampled from Normal distribution with mean 0.5 and standard deviation 0.01):

  $\lambda_{22} = 0.493, \lambda_{23} = 0.512, \lambda_{24} = 0.514, \lambda_{25} = 0.503, \lambda_{26} = 0.507, \lambda_{27} = 0.498, \lambda_{28} = 0.488, \lambda_{29} = 0.495, \lambda_{30} = 0.482$.

D Experimental Settings for COVID-19 Data in China

Selection of the provinces in China. Before the data is analyzed, an initial screening over provinces is applied to the COVID-19 data in China. In our study, we find this preprocessing step necessary for stability of inferring the model parameters.

Specifically, only the provinces or municipalities in China that satisfy the following conditions are considered:

1. The accumulated confirmed cases by February 10th 2020 are above 50. If the accumulated confirmed cases is too small, the newly confirmed cases touch zero frequently and the compartment model considered in this paper may not be appropriate to characterize the spread of COVID-19 in such region.

2. The estimated removal rate δ_k is not less than 10^{-10}. If the newly removed cases are too small, then the two-step procedure proposed in this model may not estimate the parameters appropriately.

3. The change of transmission parameter occurs no later than February 2nd 2020. Note that the data in China last to February 10th 2020, and the training data end on February 5th 2020. If the changing point is too late, the training data could not capture the transmission parameter in the second period.

Construction of training/validation/testing data. For COVID-19 data in China, we set $T = 31$, from January 10th to February 10th 2020. However, we remark that only the data after $T_0 = 11$-th day (January 21st) are available.

We set two thresholds, $T_{th,1} = 26$ (February 4th) and $T_{th} = 27$ (February 5th), to construct training/validation/testing data as described in Section A.1.2. Specifically, for trajectory prediction and quantitative model evaluation, the data from T_0-th day to the T_{th}-th day are used for parameter inference and the data from the $(T_{th} + 1)$-th day to the T-th day are treated as the testing set. To help choose the hyper-parameter μ, the data from the $(T_{th,1} + 1)$-th day to the T_{th}-th day are treated as the validation set. The validation error is computed by comparing the ground truth validation trajectory with the predicted one using model parameters fitted from the data from T_0-th day to the $T_{th,1}$-th day.

Choice of T_k. Recall that T_k denotes the day when λ_k changes in the k-th region. For real-world data in China, we set T_k for Hubei and other provinces except Hubei separately, since the medical resources are overwhelmed in Wuhan at the early stage of the pandemic [55]. Specifically, we choose $T_k = 24$ for Hubei, which is the maximum point of the newly confirmed cases from the T_0-th day to the T_{th}-th day in Hubei. For all the provinces or municipalities other than Hubei, we set $T_k = 23$, which is the maximum point of the averaged newly confirmed cases over provinces and municipalities other than Hubei from the T_0-th day to the T_{th}-th day.

E Experimental Settings for COVID-19 Data in Europe

Selection of countries in Europe. There are totally $n = 11$ countries in Europe considered in this study. Some countries such as Iceland and Luxembourg are excluded since their total population is too small, and some other countries like Sweden, Holland, and England are ignored due to that the removed cases in these countries are zero (from the data sources [53]) and thus the two-step procedure for parameter estimation in this paper may not be applied.

Preprocessing of the data. Since the original data oscillate much, especially over the weekends, the data are first smoothed by taking a simple moving average (which treats the unweighted average of data in 7 days around the i-th day as the new data point for the i-th day). The original data set contains the accumulated confirmed and removed cases from May 1st to August 31, 2020, lasting over 123 days. After the smoothing process which removes the first and last 3 days, the preprocessed data has $T = 116$ days in total.

Construction of training/validation/testing data. We consider $T = 116$ days of COVID-19 data in total and set two thresholds $T_{th,1} = 90, T_{th} = 105$. Then, for evaluating fitting and generalization performance, data of the first T_{th} days are used to fit the parameters and compute training errors, and the last $T - T_{th}$ days are for prediction and testing error computation. Additionally, for choosing hyper-parameter μ, the validation errors are computed by comparing the data from the $(T_{th,1} + 1)$-th day to T_{th}-th day with the predicted trajectories using parameters fitted on the data of the first $T_{th,1}$ days. Then, the choice of μ is based on validation errors.

Choice of T_k. From the observation of newly confirmed cases in the first T_{th} days, the transmission rates increase after some threshold in the selected countries in Europe. Thus, we still allow $\{\lambda_k\}$ to be time-varying for this data set as described in Section 4.2. Since there is no overwhelming strain on the selected countries' medical resources during the time considered in this study, for simplicity we set T_k to be the same for all the n countries. To be specific, we choose $T_k = 58$ for $k = 1, \ldots, n$, which is the minimum point of the averaged newly confirmed cases over countries in the first T_{th} days.

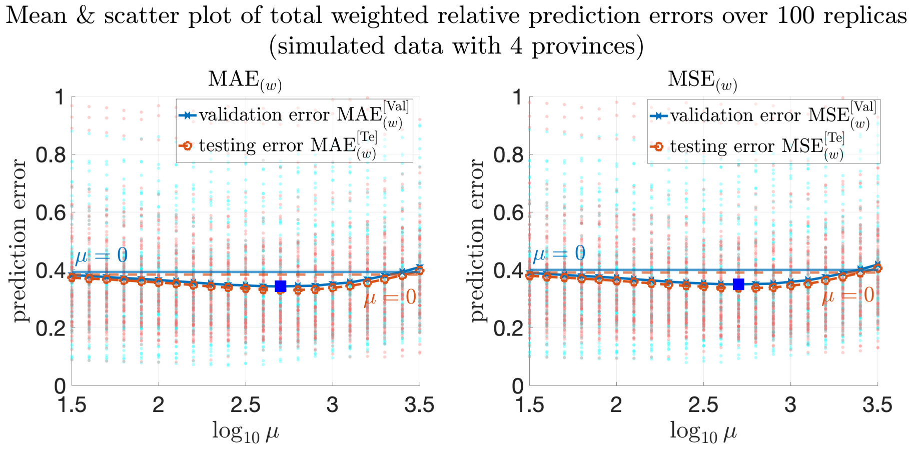

Supplementary Figure S2: Weighted testing and validation errors on simulated data with four provinces. Left: The weighted prediction errors on validation set ($MAE^{[Val]}_{(w)}$) and testing set ($MAE^{[Te]}_{(w)}$) respectively, plotted vs. the values of μ. The blue sold line and red dashed line plot the averaged errors over 100 replicas of experiment. The blue and red scatter plots show the values of the errors from the 100 replicas. Right: Same plot of MSE error. In each plot, the blue and red horizontal lines show the values of the averaged errors when $\mu = 0$. Both the MAE and MSE validation errors are minimized at $\mu = 10^{2.7}$, which is marked by yellow pentagrams in both plots. The construction of training/validation/testing data is detailed in Section A.1.1 of Supplementary Information, and the formulas of computing the errors can be found in Section B of Supplementary Information.

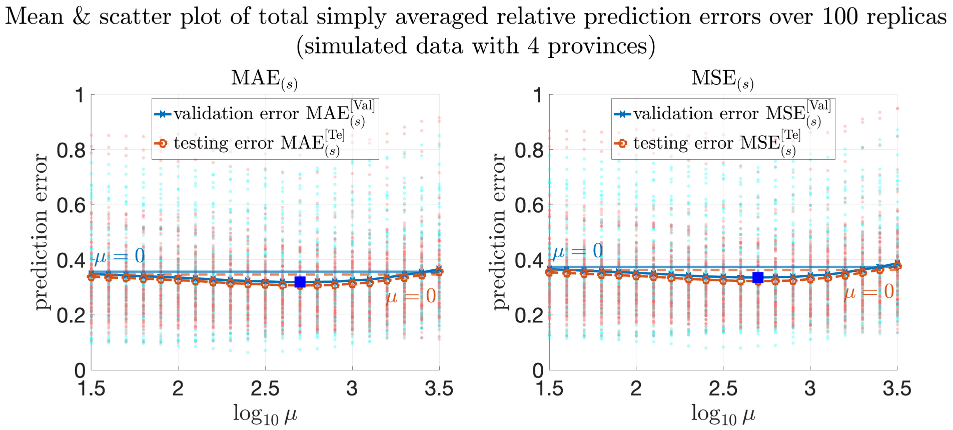

Supplementary Figure S3: Simply averaged testing and validation errors on simulated data with four provinces. The two plots are similar to those in Supplementary Figure S2, with the total errors computed as the simply averaged daily relative errors instead of the weighted daily relative errors. Note that both the MAE and MSE validation errors are still minimized at $\mu = 10^{2.7}$, which is marked by blue squares in both plots.

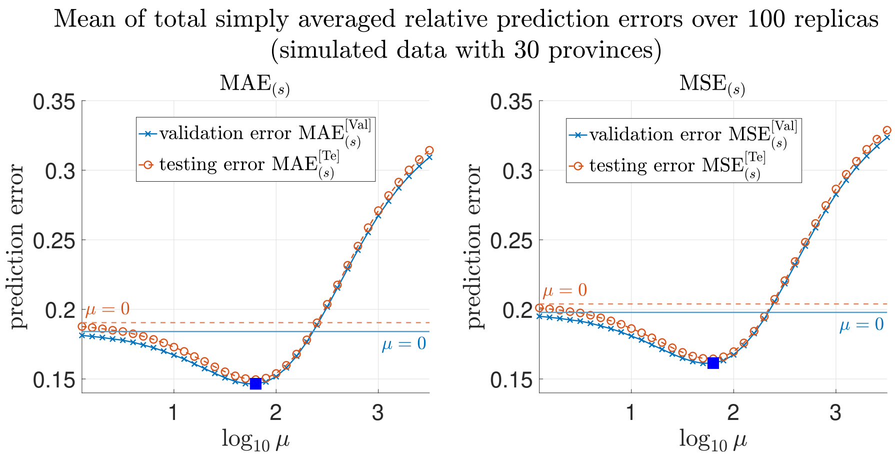

Supplementary Figure S4: Simply averaged testing and validation errors on simulated data with thirty provinces. The two plots are similar to those in Figure 7, with the total errors computed as the simply averaged daily relative errors instead of the weighted daily relative errors. Both the MAE and MSE validation errors are still minimized at $\mu = 10^{1.8}$, which is marked by blue squares in both plots.

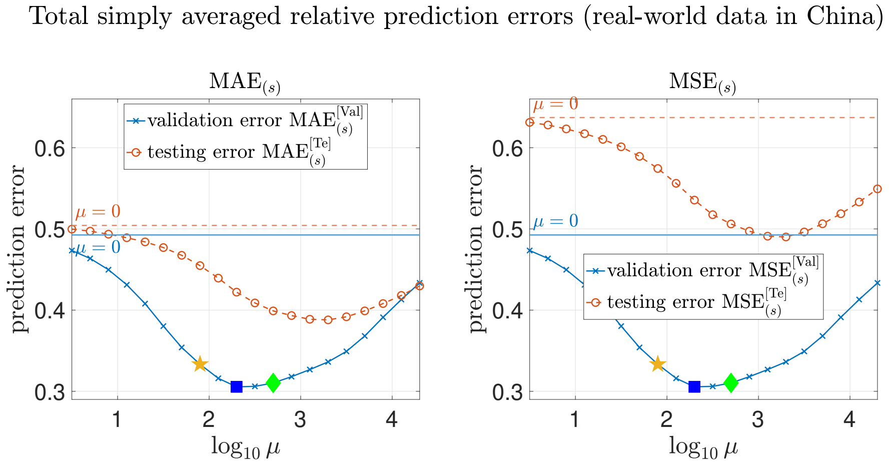

Supplementary Figure S5: Simply averaged testing and validation errors on real-world COVID-19 data in China. The two plots are similar to those in Figure 12, with the total errors computed as the simply averaged daily relative errors instead of the weighted daily relative errors. The choice of $\mu = 10^{1.9}, 10^{2.3}$, and $10^{2.7}$ has been explained in Figure 12. Note that $\mu = 10^{1.9}, 10^{2.3}$, and $10^{2.7}$ are marked by yellow pentagrams, blue squares, and green diamonds in both plots.

Total simply averaged relative prediction errors (real-world data in Europe)

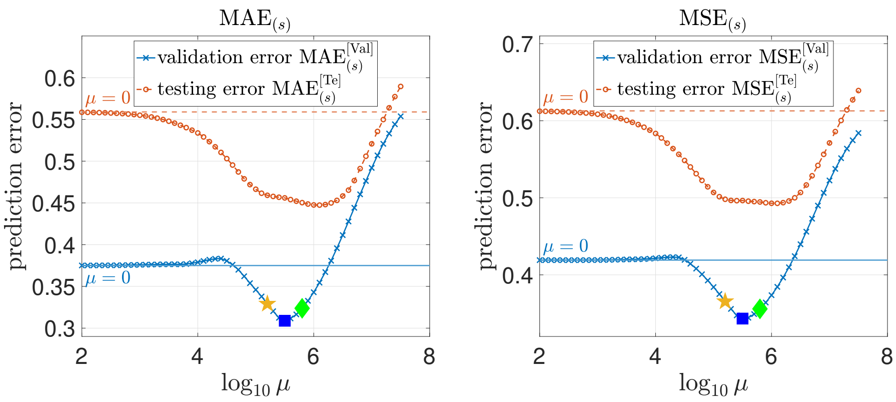

Supplementary Figure S6: Simply averaged testing and validation errors on real-world COVID-19 data in Europe. The two plots are similar to those in Figure 17, with the total errors computed as the simply averaged daily relative errors instead of the weighted daily relative errors. The choice of $\mu = 10^{5.2}, 10^{5.5}$, and $10^{5.8}$ has been explained in Figure 17. Note that $\mu = 10^{5.2}, 10^{5.5}$, and $10^{5.8}$ are marked by yellow pentagrams, blue squares, and green diamonds in both plots.
